# Highly Potent and Subtype-Selective Sperm-Specific Potassium Channel SLO3 Inhibitors Display High Tissue Exposure in the Murine Female Reproductive Tract

**DOI:** 10.1021/acsptsci.5c00416

**Published:** 2025-08-15

**Authors:** Kayla J. Temple, Ping Li, Hallie G. McKinnie, Analisa Thompson Gray, Jeanette L. Bertron, Anna E. Ringuette, Pedro de Andrade Horn, Maximilian D. Lyon, Shweta Bhagwat, Leila Asadi, Sophia Li, Roman M. Lazarenko, Ronald McCarthy, Sichen Chang, Jeremy A. Turkett, Valerie Kramlinger, Katherine J. Watson, Irene A. Zagol-Ikapitte, Jerod S. Denton, Celia M. Santi, Carrie K. Jones, Craig W. Lindsley, Olivier Boutaud

**Affiliations:** † Warren Center for Neuroscience Drug Discovery, 5718 Vanderbilt University, Nashville, Tennessee 37232, United States; ‡ Department of Pharmacology, Vanderbilt University School of Medicine, Nashville, Tennessee 37232, United States; § Department of Obstetrics and Gynecology, 12275 Washington University School of Medicine, St. Louis, Missouri 63110, United States; ∥ Department of Anesthesiology, Vanderbilt University Medical Center, Nashville, Tennessee 37232, United States; ⊥ Vanderbilt Institute of Chemical Biology, Vanderbilt University, Nashville, Tennessee 37232, United States; # Vanderbilt Brain Institute, Vanderbilt University School of Medicine, Nashville, Tennessee 37232, United States; ∇ Department of Chemistry, Vanderbilt University, Nashville, Tennessee 37232, United States; ○ Department of Biochemistry, Vanderbilt University, Nashville, Tennessee 37232, United States

**Keywords:** SLO3 inhibitors, contraception, drug metabolism, pharmacokinetics, exposure, drug distribution

## Abstract

To date, the study of drug exposure and distribution throughout the female reproductive tract has often been overlooked and neglected. Herein, we report the first highly potent and subtype-selective sperm-specific potassium channel 3 (SLO3) inhibitors as a new modality of contraceptives. After extensive *in vitro* and *in vivo* drug metabolism and pharmacokinetic profiling we selected **VU6032735** to utilize as a tool compound. **VU6032735** displayed low observed clearance with a long half-life and a high volume of distribution in female mice, which is seemingly an ideal target profile for a nonhormonal contraceptive. After intraperitoneal injection, we evaluated the drug exposure and distribution throughout various compartments of the murine female reproductive tract. Over a 24-h time course, **VU6032735** sustained high tissue exposure in the oviduct, where fertilization takes place. This work provides the framework for others interested in studying drug exposure and distribution throughout the female reproductive tract.

## Graphical abstract

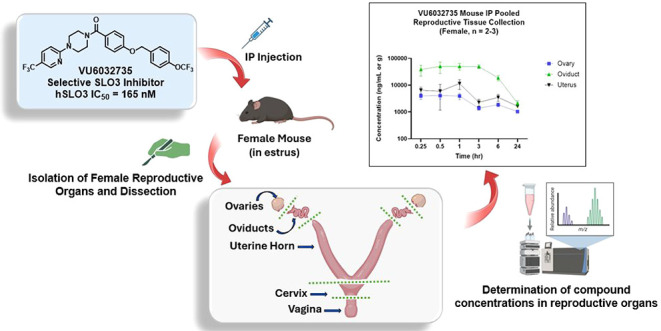

In the United States, roughly 45% (40% worldwide) of pregnancies are unintended.^1^ This high rate has been attributed to inadequate access to or incorrect use of available contraceptives. While the typical female birth control pill is 93–99% effective at preventing pregnancy, this only holds true when used perfectly.^2^ The main reason this method of contraception fails is due to inconsistent or incorrect use such as missing a dose; however, there are several other factors that are often not considered by the general populace. These include but are not limited to vomiting and/or diarrhea and drug–drug interactions (e.g., antibiotics such as Rifampin, antifungals such as Griseofulvin, certain HIV and antiseizure medicines, and the herbal remedies such as St. John’s Wort).^3^ Other slightly more invasive but highly effective (>99%) female forms of contraception include long-acting reversible contraceptives (LARCs) such as intrauterine devices (IUDs) and implants; however, like birth control pills, many of these methods are hormonal in nature and have side effects such as headaches, nausea, irregular menses, ovarian cysts, weight gain, acne, mood changes, thromboembolism, and abdominal and breast pain.^4–7^ Although rare, other possible risks associated with IUDs include irregular bleeding, an increased risk of ectopic pregnancy, bacterial infection, and the possibility of puncturing the uterine wall. Although several highly effective contraceptive methods exist, their universal acceptance has been hindered by concerns regarding such side effects as well as their convenience.^5^ Many women avoid or discontinue use of hormonal birth control methods because of their side effects; thus, there is a need to develop a novel, nonhormonal contraceptive.

One therapeutic avenue is targeting the complex maturation process, known as capacitation, of sperm. Capacitation is a process that occurs postejaculation within the female reproductive tract (FRT) and ultimately results in two key physiological changes essential for fertilization: sperm hyperactivity and acrosomal exocytosis.^8–11^ A key component of sperm capacitation is the hyperpolarization of the plasma membrane (becoming more negatively charged on the inside) upon entering the FRT.^12,13^ In fact, a frequent characteristic of men experiencing subfertility is sperm that lack adequate negative membrane potential and this has been correlated to low fertilization rates in *in vitro* fertilization (IVF).^14–16^

Potassium (K^+^) permeability is one of the key factors responsible for controlling sperm membrane potential and hyperpolarization.^13,17,18^ An emerging target in this field is the sperm-specific potassium channel (SLO3), which is crucial for normal sperm functions that are required for fertilization including sperm hyperpolarization and capacitation in both mice and humans.^19,20^ In fact, male knockout mice lacking SLO3 are infertile as are males with a genetic mutation in the Potassium Calcium-Activated Channel Subfamily U Member 1 gene (KCNU1), which encodes the SLO3 channel in humans.^11,21–24^ Therefore, it is critical to identify potent and selective SLO3 inhibitors that could be developed as novel nonhormonal contraceptive agents. Our efforts have focused on developing compounds with increased selectivity for SLO3 over other members of the SLO potassium (K^+^) channel family, SLO1 and SLO2, which are broadly expressed in the body, as well as over the human voltage-sensitive K^+^ human ether-a-go-go channel (hERG), a known off-target with essential roles in cardiac repolarization.^25–27^ Recent work from the Santi Lab using **VU0546110**, a subtype-selective SLO3 inhibitor identified in a high-throughput screen at Vanderbilt University (human SLO3 IC_50_ = 1.29 μM), demonstrated that SLO3 is the sole K^+^ channel responsible for hyperpolarization and fertilizing capacity in human sperm.^28^ While **VU0546110** exhibits a 46-fold selectivity for SLO3 over SLO1, its selectivity over hERG is limited to just 1.5-fold, raising concerns about potential cardiotoxicity. Therefore, our next objective was to develop compounds with improved human SLO3 (hSLO3) potency devoid of hERG liabilities.

Utilizing the same high-throughput screening (HTS) method as previously described, we identified two additional chemical scaffolds: **VU0528254** (hSLO3 IC_50_ = 223 nM) and **VU0530373** (hSLO3 IC_50_ = 445 nM) (Figure 1). Although both HTS hits displayed submicromolar SLO3 potency, they suffered from the same issue as **VU0546110** as neither **VU0528254** (4.2-fold selective) nor **VU0530373** (1.1-fold selective) was selective over hERG. Our goal was to develop a compound with improved human SLO3 potency (hSLO3 IC_50_ < 200 nM), hERG selectivity (≥100-fold), and SLO1/2 selectivity (≥20-fold) that could be utilized as an *in vivo* tool to study drug exposure in the FRT of rodents. This work details the drug metabolism and pharmacokinetic profiles of two such compounds, **VU6032735** and **VU6047606** (a diastereomer of **VU6045330**), as well as their exposures throughout the female reproductive tract.

**Figure 1:**
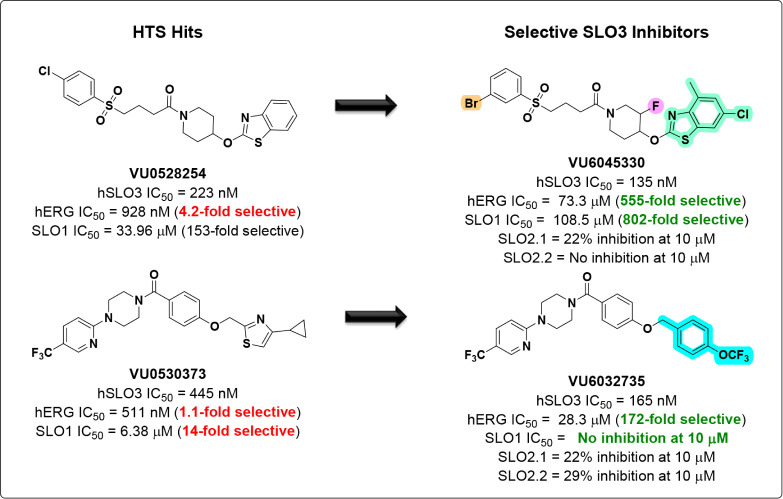
Chemical optimization of two unique scaffolds identified in a high-throughput screen resulted in the discovery of highly potent and selective SLO3 inhibitors.

## Results

### Identification of Highly Potent SLO3 Inhibitors Lacking hERG Liabilities

Following parallel iterative synthesis utilizing the HTS hit **VU0528254** as a starting point, we generated the analog **VU6045330** (Figure 1). **VU6045330** displayed improved human SLO3 potency (hSLO3 IC_50_ = 135 nM) when compared to the predecessor analog **VU0528254** (hSLO3 IC_50_ = 223 nM). Next, we assessed the hERG liability of **VU6045330**, which revealed a dramatically improved hERG selectivity profile (hERG IC_50_ = 73.3 μM, 555-fold selectivity) when compared to the analog **VU0528254** (hERG IC_50_ = 928 nM, 4.2-fold selectivity) (Figure 1).

As **VU6045330** was originally synthesized as a mixture of four diastereomers, we elected to synthesize the four diastereomerically pure isomers before further *in vitro* and *in vivo* pharmacokinetic (PK) characterization. To this end, we synthesized isomers **VU6047606**, **VU6047607**, **VU6047608**, and **VU6047609**. While all four isomers were submicromolar inhibitors of human SLO3, only **VU6047606** and **VU6047609** were highly potent inhibitors of human SLO3 (IC_50_ *s* ≤ 112 nM) (Figure 2A). Upon further investigation, we confirmed that **VU6047606, VU6047607**, and **VU6047609** were highly selective for SLO3 versus hERG (≥130-fold) while **VU6047608** was only moderately selective (37-fold) (Table 1). In a similar manner, we employed the HTS hit **VU0530373** as a starting point, which led to the identification of **VU6032735** (Figure 1). **VU6032735** displayed a nearly 3-fold improvement in potency (hSLO3 IC_50_ = 165 nM) versus its predecessor analog **VU0530373** (hSLO3 IC_50_ = 445 nM) (Figure 2B).^29^ Moreover, **VU6032735** was 172-fold selective for SLO3 compared to hERG, which was a substantial improvement over **VU0530373** (1.1-fold selectivity) (Table 2).

**Figure 2:**
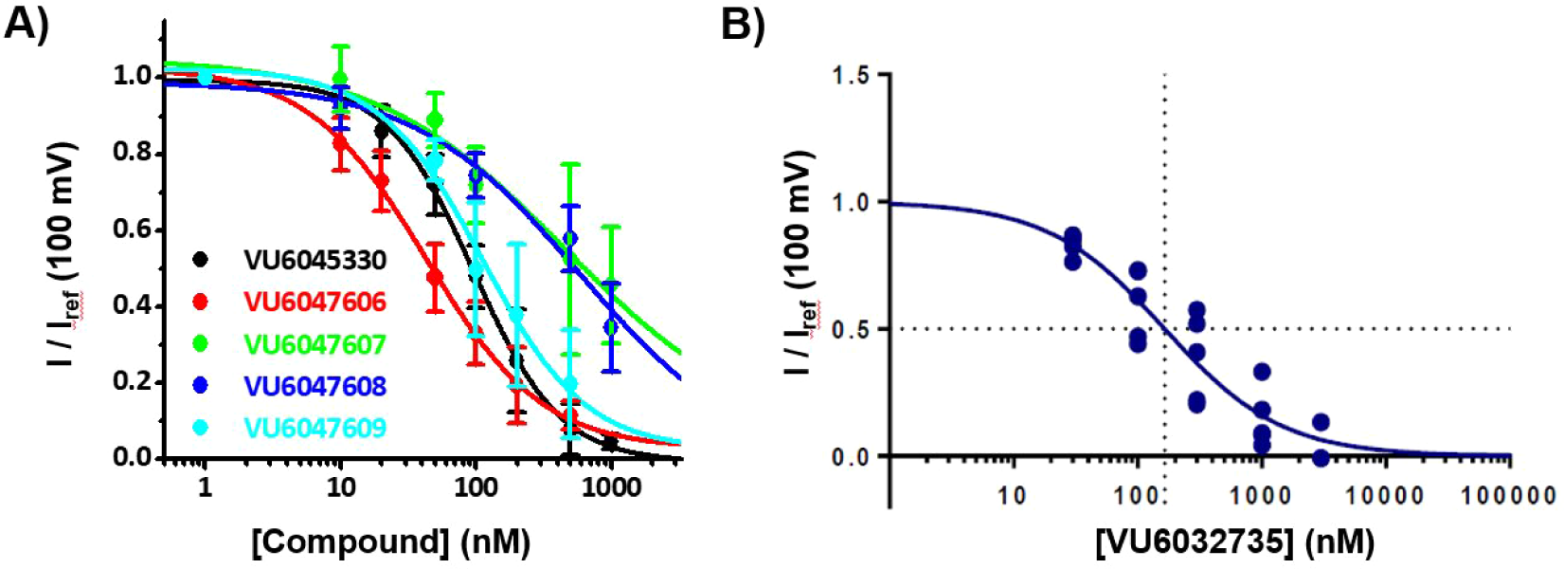
Dose-response curves from human SLO3-γ2 expressed in HEK cells of (**A**) **VU6045330** and its four diastereomerically pure isomers and (**B**) **VU6032735**. Currents at +100 mV were normalized to the current with no compound and fit with a modified Hill equation to derive the IC_50_ (*n* = 5–6). Data are represented as mean ± SD.

**Table 1:**
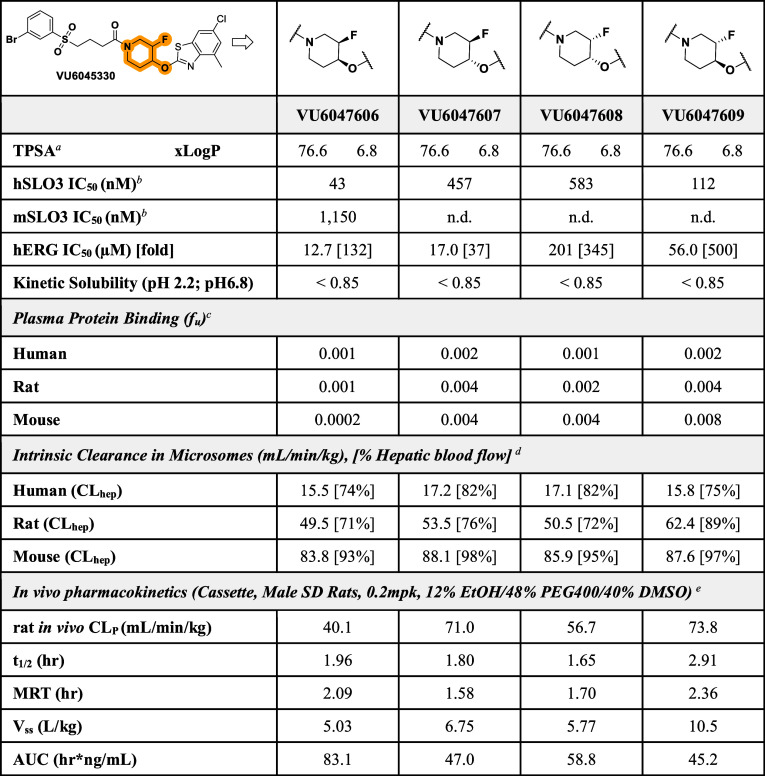
Key Properties and Pharmacokinetic Parameters of Diastereomerically Pure Isomers of **VU6045330**

a TPSA = total polar surface area.

b Potency was determined in transfected HEK293 cells.

c *f*_*u*_ = Fraction unbound; equilibrium dialysis assay.

d CL_hep_ = *in vitro* hepatic clearance.

e CL_p_ = *in vivo* plasma clearance, *t*_1/2_ = elimination half-life, MRT = mean retention time, *V*_ss_ = volume of distribution, AUC = area under curve.

**Table 2:**
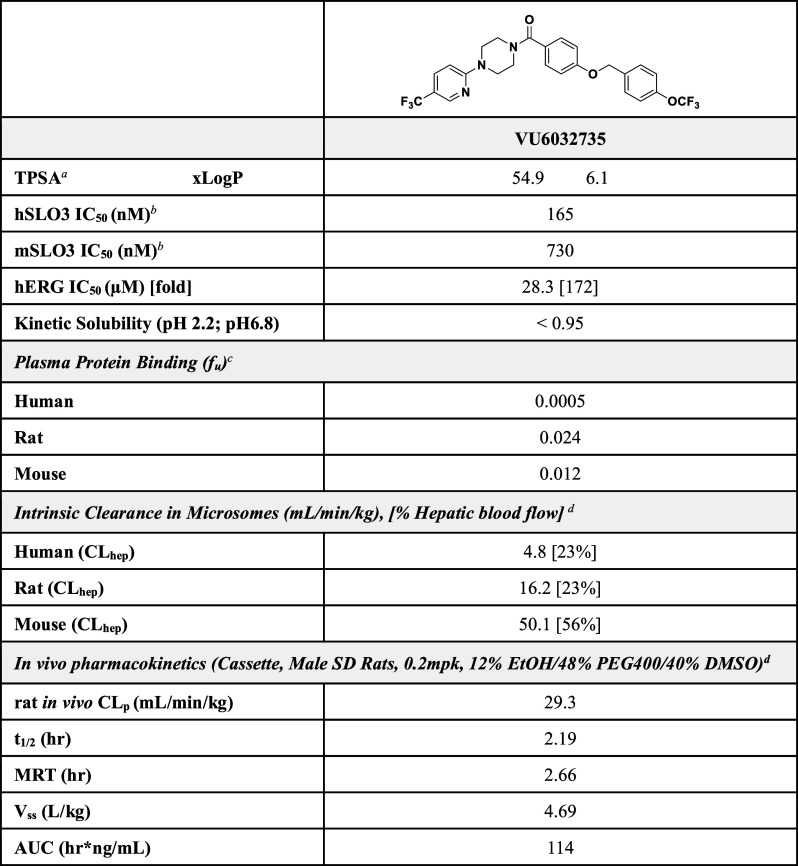
Potency, Selectivity, and Pharmacokinetic Parameters of Diastereomerically Pure Isomers of **VU6032735**

a TPSA = total polar surface area.

b Potency was determined in transfected HEK293 cells.

c *f*_*u*_ = Fraction unbound; equilibrium dialysis assay.

d CL_hep_ = *in vitro* hepatic clearance.

e CL_p_ = *in vivo* plasma clearance, *t*_1/2_ = elimination half-life, MRT = mean retention time, *V*_ss_ = volume of distribution, AUC = area under curve.

### *In Vitro* Assessment of Plasma Protein Binding and Hepatic Clearance

To select which diastereomer to carry forward *in vivo*, we first evaluated *f*_*u*_ in human, rat, and mouse plasma (Table 1). Unfortunately, all four diastereomers exhibited high plasma protein binding (*f*_*u*_ < 1%). To differentiate the diastereomers, we next examined hepatic clearance (CL_hep_) using human, rat, and mouse microsomes. All four diastereomers displayed high human (CL_hep_ > 15 mL/min/kg), rat (CL_hep_ > 46 mL/min/kg), and mouse (CL_hep_ > 60 mL/min/kg) hepatic clearance based on microsomal intrinsic clearance (CL_int_) data.

Similarly, we examined *f*_*u*_ and CL_hep_ of **VU6032735** in human, rat, and mouse (Table 2). While **VU6032735** displayed high plasma protein binding in human , a moderate fraction unbound was observed in rat (*f*_*u*_ = 2.4%) and mouse (*f_u_* = 1.2%). Unlike the first chemical series, **VU6032735** demonstrated low hepatic clearance in human (CL_hep_ = 4.8 mL/min/kg) and rat (CL_hep_ = 16.2 mL/min/kg), while mouse hepatic clearance was moderate (CL_hep_ = 50.1 mL/min/kg).

### *In Vivo* Assessment of PK in Rats

To quickly ascertain whether our two novel chemical series suffered from an *in vitro*–*in vivo* correlation disconnect, we evaluated all five compounds in intravenous pharmacokinetics (IVPK). For these studies, we chose to examine the PK in rats as they are often used for toxicology evaluations, and we were able to evaluate our compounds utilizing a cassette format. When assessed *in vivo*, **VU6032735** displayed a moderate plasma clearance (CL_p_ = 29.3 mL/min/kg) with an elimination *t*_1/2_ of 2.19 h and a volume of distribution (*V*_ss_) of 4.69 L/kg (Table 2). While three of the four diastereomers displayed high plasma clearance in rats, **VU6047606** demonstrated moderate plasma clearance (CL_p_ = 40.1 mL/min/kg) with an elimination half-life (*t*_1/2_) of 1.96 h and a volume of distribution (*V*_ss_) of 5.03 L/kg (Table 1). For this reason, **VU6047606** was selected as the diastereomer to carry forward for further profiling.

### Evaluating Inhibition of Cytochrome P450 (CYP_450_), hSLO1, hSLO2.1, and hSLO2.2

With two compounds of interest in hand, further studies were conducted to identify which analog would provide the best *in vivo* tool compound. To this end, we first evaluated the CYP_450_ inhibition profiles of **VU6032735** and **VU6047606**. Both analogs showed >30 μM inhibition of all CYP isoforms tested (CYP1A2, CYP2D6, CYP2C9, and CYP3A4) (Table 3). Next, we assessed the selectivity profiles of both analogs in relation to SLO1 and SLO2 (Table 3). **VU6032735** displayed selectivity for hSLO3 versus hSLO1 (no inhibition at 30 μM), hSLO2.1 (22%inhibition at 10 μM), and hSLO2.2 (29% inhibition at 10 μM). Likewise, **VU6047606** displayed selectivity for hSLO3 versus hSLO1 (10% inhibition at 30 μM) and hSLO2.1 (4% inhibition at 10 μM).

**Table 3:** Further Evaluations of **VU6047606** and **VU6032735** as Inhibitors of hSLO1, hSLO2, and CYP P450s

Compound	HEK hSLO3% of inhib. at 10 μM	HEK hSLO1% inhib. at 30 μM)	HEK hSLO2.1% inhib. 10 μM)	HEK hSLO2.2% inhib. 10 μM)	CYP_450_ Inhibition (μM) (1A2, 2D6, 2C9, 3A4)
**VU6047606**	100%	10%	4%	n.d.	>30
**VU6032735**	100%	No inhibition	22%	0%	>30

### Evaluation of Human Sperm Hyperactivated Motility

Upon entering the FRT, sperm undergo a physiological transformation known as capacitation, which is essential for fertilization. One measurable phenomenon of capacitation is the development of sperm hyperactivated motility. To determine the effects of **VU6032735** and **VU6047606** have on hyperactivated motility, we incubated human sperm in capacitating conditions for 2 h followed by incubation for 1 min (acute) with 10 μM of either **VU6032735** or **VU6047606**. Both **VU6032735** (76% inhibition) and **VU6047606** (71% inhibition) significantly inhibited human sperm hyperactivated motility when acutely dosed (Figure 3). To determine if the inhibitory effects of our analogs are acute and diminish over time (like the previously reported **VU0546110**), sperm were incubated for 2 h in capacitation media with 10 μM of either **VU6032735** or **VU6047606**. Interestingly, inhibition of hyperactivated motility was still observed with both **VU6032735** (50% inhibition) and **VU6047606** (61% inhibition).^28^

**Figure 3:**
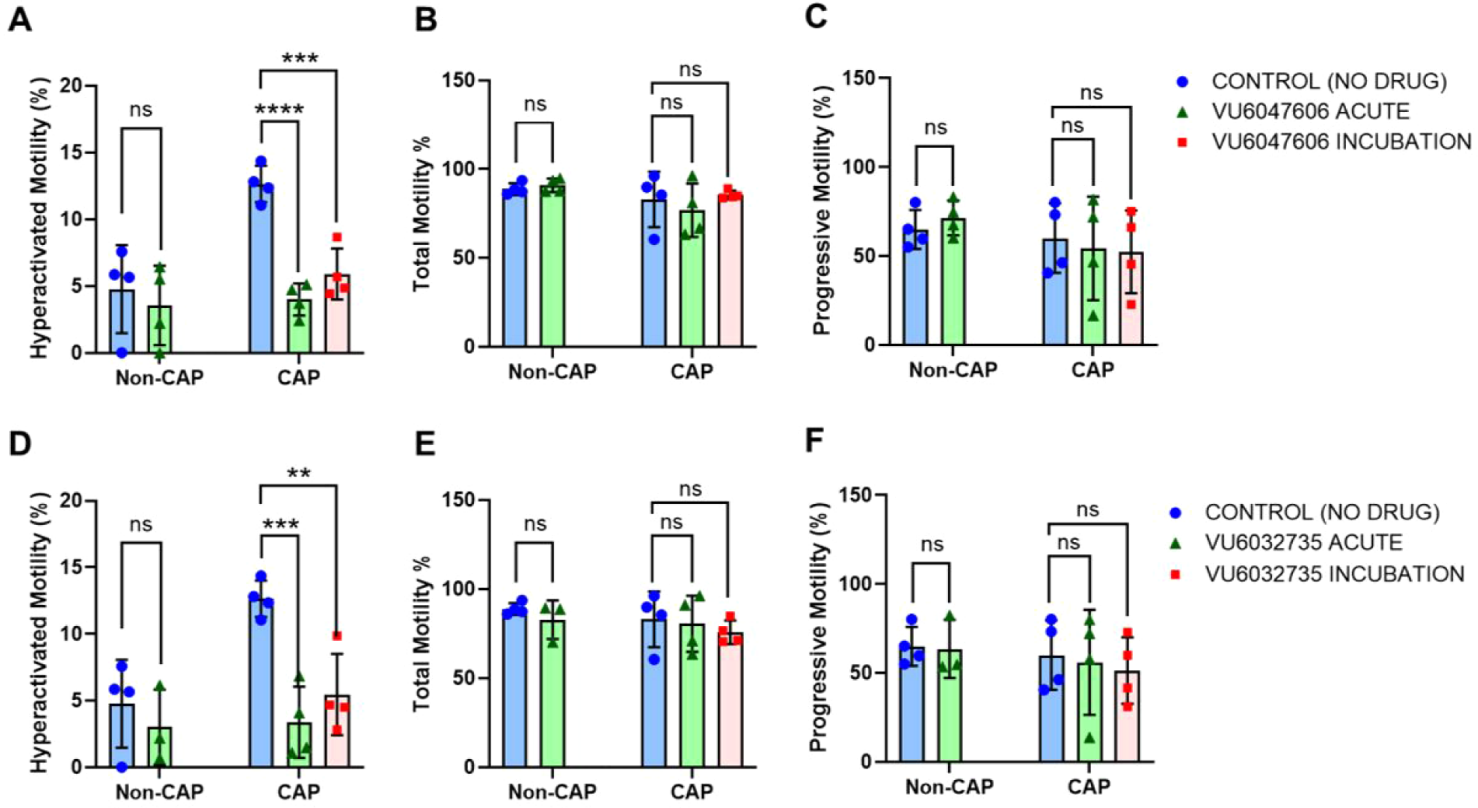
**VU6047606** and **VU6032735** significantly inhibit human sperm hyperactivated motility at 10 μM. CASA measurements were obtained for hyperactivated motility (A,D), total motility (B,E), and progressive motility (C,F) with human sperm incubated under noncapacitating (Non-CAP) and capacitating (CAP) conditions in the presence and absence of 10 μM **VU6047606** (A,B,C), or **VU6032735** (D,E,F), (*n* = 4). “Blue bars” represent media controls, “green bars” represent acute inhibitor exposure, and “red bars” indicate sperm incubation with the inhibitors for 120 min under CAP conditions. Data were analyzed by two-way ANOVA with Bonferroni’s multiple comparison test and are presented as mean and standard deviation. **p < 0.01, ***p < 0.001, ****p < 0.0001, ns = nonsignificant. Data are represented as mean ± SD.

### Determining Mouse Potency of SLO3 Expressed in HEK293 Cells and Mouse Spermatozoa^29^

As the SLO3 mechanism has been extensively studied in mice, we evaluated the mouse SLO3 (mSLO3) potency of both **VU6032735** and **VU6047606** in preparation for future murine studies. First, inhibitory activity was assessed in HEK293 cells transfected with murine SLO3-γ2 (Figure 4A). While both **VU6032735** (mSLO3 IC_50_ = 730 nM) and **VU6047606** (mSLO3 IC_50_ = 1.15 μM) inhibited mouse SLO3 currents, **VU6032735** was ∼1.6-fold more potent. Similarly, whole-cell recordings from mouse sperm demonstrated that **VU6032735** (mSLO3 IC_50_ = 910 nM) was ∼1.6-fold more potent than **VU6047606** (mSLO3 IC_50_ = 1.47 μM) at inhibiting mouse SLO3 currents (Figure 4B). These results indicate that both **VU6032735** (∼16-fold) and **VU6047606** (∼7-fold) are less potent on the murine SLO3 channel when compared to the human SLO3 channel, which is likely due to SLO3 showing only ∼60% sequence homology between mouse and human.^20^

**Figure 4:**
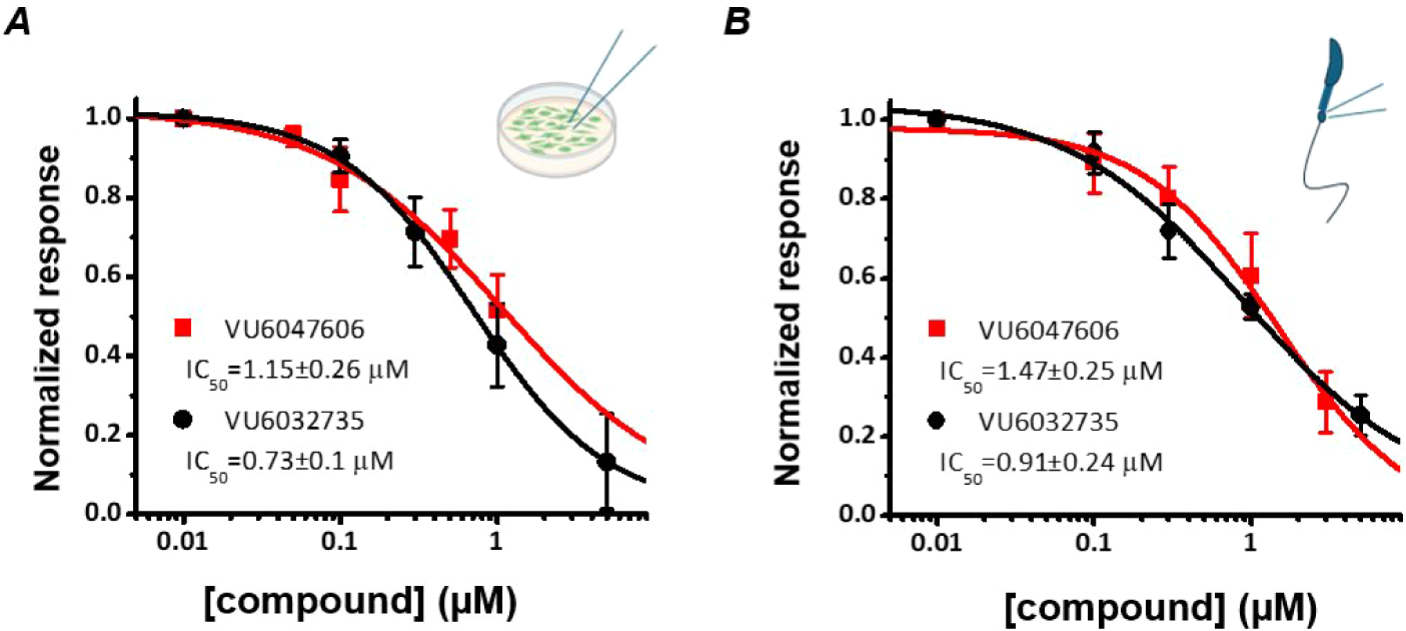
Dose-response curves of **VU6047606** and **VU6032735** from mouse SLO3-γ2 expressed in HEK293 cells **(A)** or mouse sperm **(B)**. Currents at +100 mV were normalized to the current in the absence of the compound and fitted with a modified Hill equation to derive IC_50_ values. **VU6047606** inhibits mouse SLO3-γ2 channels with an IC_50_ = 1.15 ± 0.26 μM and Hill coefficient = 0.83 ± 0.19 (*n* = 5). **VU6032735**’s IC_50_ = 0.73 ± 0.10 μM, Hill coefficient = 1.03 ± 0.02 (*n* = 5). **VU6047606** inhibits mouse KSper currents with IC_50_ = 1.47 ± 0.25 μM, Hill coefficient = 1.02 ± 0.12 (*n* = 5); **VU6032735** inhibits mouse KSper currents with IC_50_ = 0.91 ± 0.24 μM, Hill coefficient = 0.78 ± 0.07 (*n* = 5). Data are represented as mean ± SD.

### Multispecies Metabolite Identification (MetID)

After a 4-h incubation period, **VU6032735** showed minimal metabolism with >98% of the parent compound remaining in human, rat, mouse, dog, cynomolgus monkey, and minipig hepatocytes.^29^ Although all metabolites were <0.5% in abundance, mouse, rat, dog, and cynomolgus monkey provided full coverage for observed human metabolites with no human-specific metabolites. Undergoing the same incubation conditions, **VU6047606** displayed minimal metabolism across species with >82% of the parent compound remaining in human, rat, mouse, dog, cynomolgus monkey, and minipig hepatocytes (Figure 5).^29^ The major metabolite, **M781b**, showed a 6% relative abundance and was the result of glucuronidation. It was also noted that no human-specific metabolites were observed, with mouse, rat, minipig, and cynomolgus monkey providing full coverage across all observed human metabolites.

**Figure 5:**
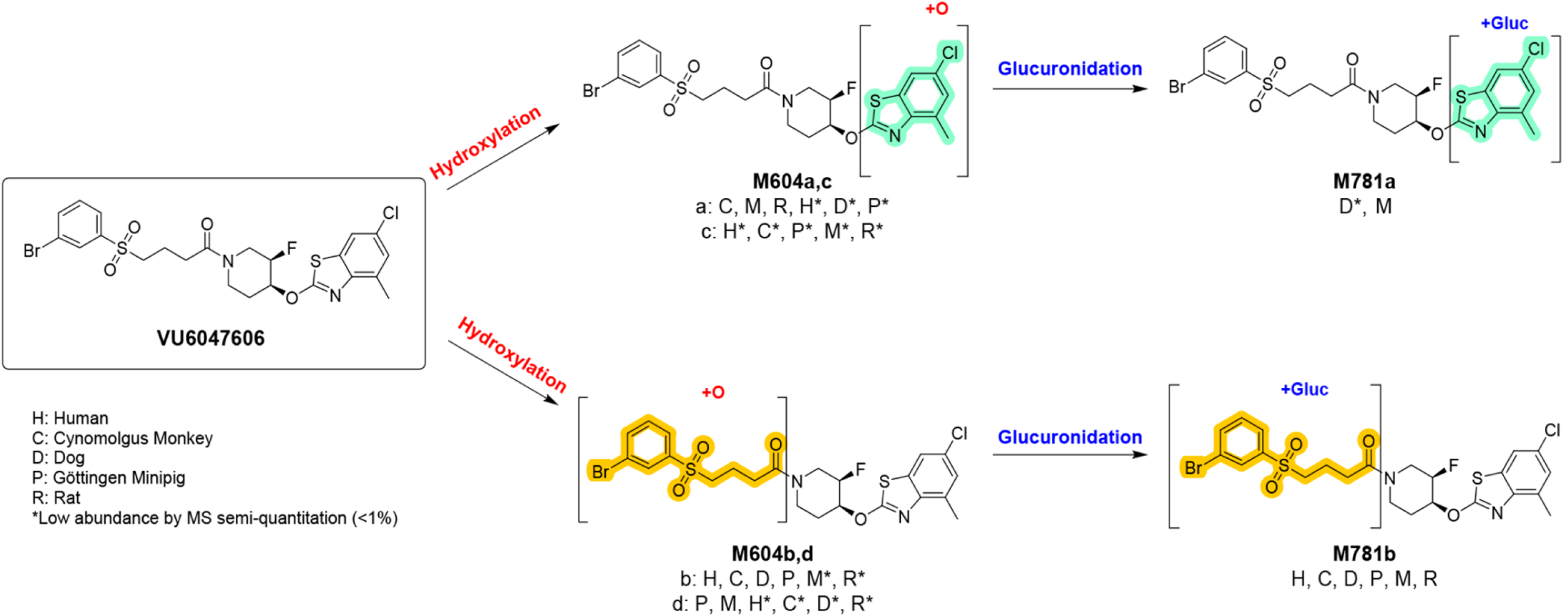
Multi-species MetID study shows minimal metabolism of **VU6047606** after 4 h incubation with hepatocytes with hydroxylation and glucuronidation being the major metabolic pathways.

### Off-Target Safety/Toxicity Profiles

As these were novel chemical scaffolds, we were interested in investigating the off-target and safety and toxicity profiles of **VU6032735** and **VU6047606**. In a LeadProfilingScreen employing radioligand displacement to evaluate ancillary pharmacology, a panel of 68 potential off-target G protein-coupled receptors, neuronal hormone receptors, ion channels, and transporters was screened. Compounds were screened at 10 μM and percent inhibition was evaluated. **VU6047606** showed 83% inhibition of the sodium channel, site 2 as well as 93% inhibition of the L-type calcium channel (also known as the dihydropyridine channel). Similarly, **VU6032735** displayed 81% inhibition of the sodium channel, site 2 as well as 76% inhibition of the L-type calcium channel.^29^

### Evaluating the Drug Concentration Ratio between Murine Female Reproductive Organs and Plasma after IV Dosing

Both **VU6032735** and **VU6047606** were dosed at 1 mg/kg IV in female mice and samples were collected at the 15-min time point to determine drug concentrations in the plasma and the collective reproductive organs to determine the distribution ratio (Figure 6 and Table 4). **VU6047606** concentration in plasma (121 ng/mL) was higher when compared to the pooled reproductive organs (56.6 ng/mL) resulting in a reproductive organ/plasma ratio of 0.47. Conversely, **VU6032735** provided higher concentrations of drug in the pooled reproductive organs (1070 ng/mL) versus the plasma samples (85.3 ng/mL), resulting in a reproductive organ/plasma ratio of 12.7. Given the overall drug metabolism and pharmacokinetic (DMPK) profile of **VU6032735** as well as higher distribution into murine female reproductive organs, we selected **VU6032735** as our tool compound to move forward into studies evaluating the distribution of the drug into the various anatomical compartments of the female reproductive tract.

**Figure 6:**
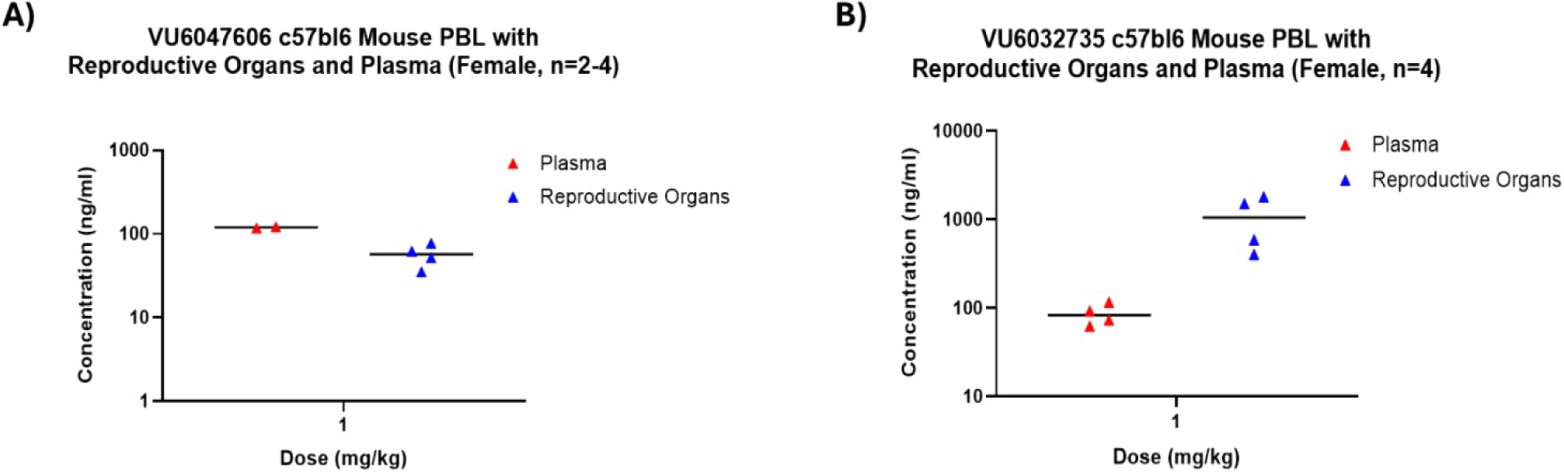
Assessment of the distribution ratio between the murine female reproductive organs and plasma after 1 mg/kg IV dosing of SLO3 inhibitors. **(A)** **VU6047606** shows higher concentrations in plasma versus female reproductive organs. **(B)** **VU6032735** displays higher concentrations in the female reproductive organs versus plasma.

**Table 4:** Drug Distribution Relationship between Plasma and Reproductive Organs after IV Dosing in Female Mice

Compound	Dose (mg/kg)	Time (hr)	Plasma (ng/mL)	Reproductive Organs (ng/mL)	Reproductive Organs/Plasma Ratio
**VU6047606**	1, IV	0.25	121	56.6	0.47
**VU6032735**	1, IV	0.25	85.3	1070	12.7

### *In Vivo* Assessment of PK Parameters in Female Mice

As mice are a species in which the SLO3 mechanism has been extensively studied, we envision future studies to include a murine pharmacodynamic assay of fertility. Therefore, we elected to evaluate **VU6032735** in discrete IV PK studies in female mice. When dosed at 5 mg/kg, **VU6032735** displayed low observed clearance (CL_obs_ = 8.6 mL/min/kg) with an elimination half-life of 33.7 h and a high volume of distribution (*V*_ss_ = 23.6 L/kg) (Table 5).

**Table 5:** IV PK Profile of **VU6032735** Dosed at 5 mg/kg in Female Mice^a^

Compound	Dose (mg/kg)	Elim. t_1/2_ (hr)	MRT (hr)	CL_obs_ (mL/min/kg)	V_ss_ (L/kg)	AUC (hr*ng/mL)
**VU6032735**	5, IV	33.7	45.9	8.6	23.6	9835

a *t*_1/2_ = elimination half-life; MRT = mean retention time; CL_obs_ = observed *in vivo* clearance; *V*_ss_ = volume of distribution; AUC = area under curve.

### Evaluating Exposure and Distribution of **VU6032735** in the Various Anatomical Compartments of the Murine Female Reproductive Tract after IP Dosing

To determine how many tissues needed to be pooled to consistently quantify exposure in the ovaries, oviducts, uterus, cervix, and vagina, **VU6032735** was dosed IP at 10 mg/kg in mice and the different compartments of the murine FRT were pooled from 1, 2, and 3 mice. Our data show that pooling tissues from 2 mice enabled us to obtain consistent quantification after IP dosing (Figure 7).

**Figure 7:**
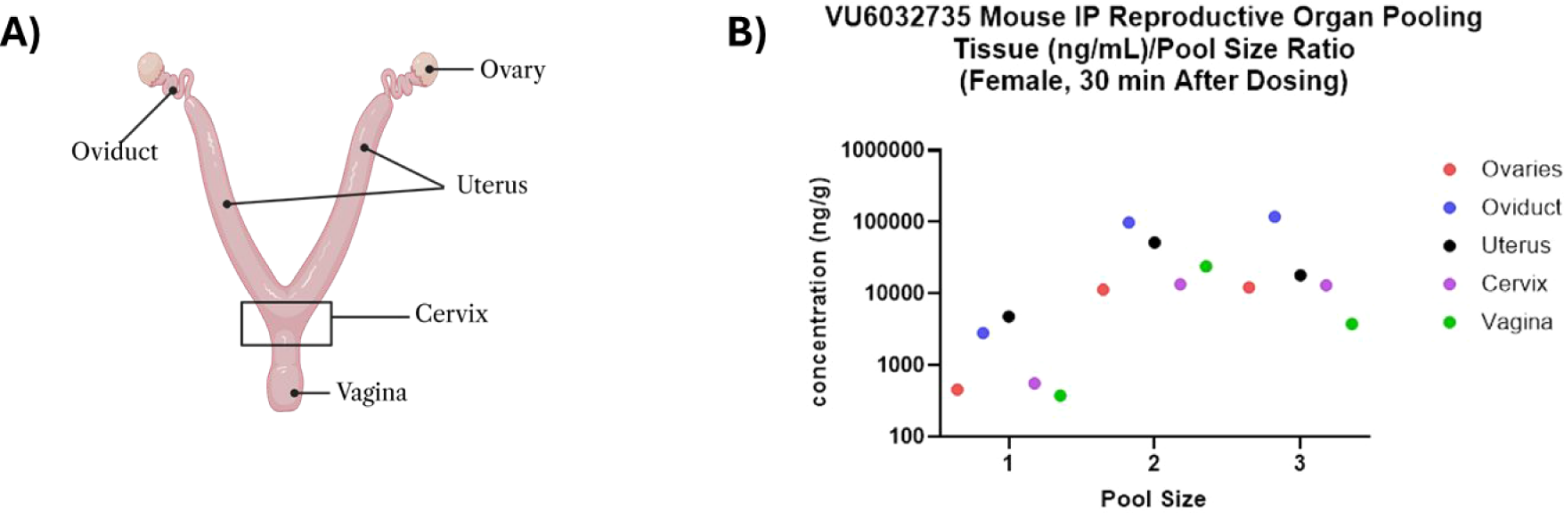
**VU6032735** was dosed at 10 mg/kg IP in female mice to determine the tissue sample/pool size needed for LC-MS/MS analysis. **(A)** The female reproductive tract was dissected into 5 compartments for analysis. **(B)** Concentration of **VU6032735** if the various anatomical compartments in relation to varying pool size (1–3 animals).

To confirm that **VU6032735** can achieve high tissue exposure for a sustained period of time, we dosed mice IP at 10 mg/kg and measured its concentration in the plasma, ovaries, oviducts, and uterus over 24 h (Figure 8). Our data show that total concentrations in the different compartments of the reproductive tract remained above 1 μM for 24 h after dosing in female mice (Table 6). In the oviduct, where fertilization occurs, total drug concentration was measured at 4.02 μM at 24 h, which is 4.4- to 5.5-fold higher than the mSLO3 IC_50_ determined utilizing mouse sperm and mouse SLO3-γ2 transfected HEK293 cells, respectively. We acknowledge that the conditions under which the IC_50_ was determined in the electrophysiology experiments—namely, noncapacitated sperm at room temperature following acute drug exposure—differ from the physiological conditions in the oviduct, where sperm are typically capacitated and at 37 °C. Therefore, future studies conducted under more physiologically relevant conditions will be necessary to refine these estimates.

**Figure 8:**
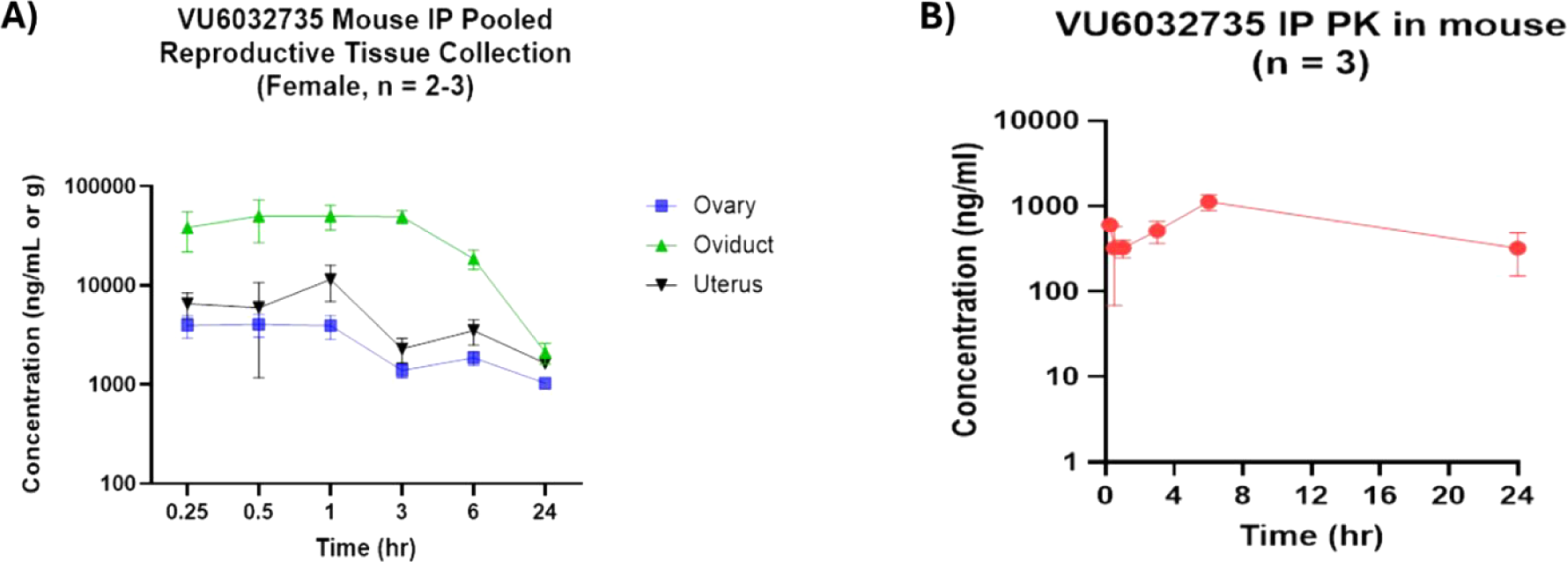
**VU6032735** was dosed at 10 mg/kg IP in mice and exposure was monitored in pooled **(A)** reproductive organs and **(B)** plasma over 24 h. Data are represented as mean ± SD.

**Table 6:** Tissue Distribution of **VU6032735** in Various Anatomical Compartments of Female Mice

Compound	Time (hr)	Plasma (μM)	Ovary (μM)	Oviduct (μM)	Uterus (μM)
**VU6032735**	0.25	1.15	7.52	73.0	12.4
	0.5	0.45	7.69	94.8	11.3
	1	0.62	7.45	95.2	21.8
	3	0.98	2.63	93.6	4.35
	6	2.14	3.54	35.3	6.66
	24	0.61	1.97	4.02	3.11

## Discussion

Herein, we have disclosed the first-ever highly potent (hSLO3 IC_50_ ≤ 165 nM) and subtype-selective SLO3 inhibitors (**VU6047606** and **VU6032735**) that also displayed high selectivity for SLO3 over hERG as well as its close relatives, SLO1 and SLO2. Additionally, when acutely dosed, both **VU6047606** and **VU6032735** significantly inhibited human sperm hyperactivated motility by >70% without changing total or progressive motility. Thus, both **VU6047606** and **VU6032735** were selected to advance into a battery of *in vitro* and *in vivo* DMPK assays to assess their suitability as potential tool compounds to study drug exposure within various tissues of the murine FRT. To our knowledge, very little research has been conducted on drug exposure within the FRT when administered via a nonvaginal route of drug delivery.

In an effort to select one compound to move forward, we first evaluated plasma protein binding and predicted hepatic clearance in various species. **VU6047606** exhibited high plasma protein binding (*f*_*u*_ < 1.0%) in all species tested (mouse, rat, and human). Similarly, **VU6032735** exhibited high plasma protein binding in human; however, moderate plasma protein binding was observed in rat (*f*_*u*_ = 2.4%) and mouse (*f_u_* = 1.2%). **VU6047606** displayed high human and rat predicted hepatic clearance based on microsomal CL_int_ data (human CL_hep_ > 15 mL/min/kg; rat CL_hep_ > 46 mL/min/kg; mouse CL_hep_ > 60 mL/min/kg). Conversely, **VU6032735** provided a more favorable *in vitro* clearance profile with low predicted hepatic clearance in both human and rat (human CL_hep_ = 4.8 mL/min/kg; rat CL_hep_ = 16.2 mL/min/kg) and moderate predicted hepatic clearance in mouse (CL_hep_ = 50.1 mL/min/kg). To ascertain if the *in vitro* clearance values were a true representation of *in vivo* clearance, we evaluated both **VU6047606** and **VU6032735** in rat IV PK using a cassette format. Here, both **VU6047606** and **VU6032735** displayed moderate plasma clearance (40.1 and 29.3 mL/min/kg, respectively) with similar *t*_1/2_ (1.96 and 2.19 h, respectively) and *V*_ss_ (5.03 and 4.69 L/kg, respectively).

To aid in tool compound selection, we next evaluated the inhibition of various CYP_450_ isoforms (CYP1A2, CYP2D6, CYP2C9, and CYP3A4). To our delight, both **VU6047606** and **VU6032735** displayed CYP_450_ IC_50_ *s* > 30 μM; however, this did not help our compound selection. Therefore, we elected to examine off-target safety profiles as well as multispecies hepatocyte MetID. Both analogs were subjected to a LeadProfilingScreen and afforded similar off-target profiles; however, **VU6047606** inhibited the calcium channel L-type, dihydropyridine (93% inhibition) more so that **VU6032735** (76% inhibition) in a radioligand binding assay. Both compounds also inhibited the sodium channel type 2 (around 80% inhibition) in a radioligand binding assay. However, the inhibition of radioligand binding does not always translate into functional inhibition. As an example, **VU6047606** at 10 μM inhibited hERG radioligand binding by 72% in this assay but the IC_50_ was determined to be 12 μM, providing a safety margin of 276-fold. Nevertheless, we will be careful to also assess selectivity toward these channels as we develop lead candidates. Both compounds also performed well in the multispecies hepatocyte MetID. After a 4-h incubation period, >82% of parent **VU6047606** remained indicating minimal metabolism in all species tested (human, rat, mouse, dog, cynomolgus monkey, and minipig). Similarly, >98% of parent **VU6032735** remained after the 4-h incubation period.

Thus far, both compounds presented very similar DMPK profiles. Therefore, we elected to carry both compounds forward into a study evaluating the drug concentration ratios between murine female reproductive organs and plasma after a 1 mg/kg IV dose. **VU6047606** afforded a reproductive organ/plasma ratio of 0.47, indicating higher drug concentrations in plasma versus female reproductive organs. Conversely, **VU6032735** displayed a reproductive organ/plasma ratio of 12.7, indicating higher drug concentrations in the female reproductive organs when compared to plasma. This key factor led us to select **VU6032735** as our tool compound to progress forward.

As future plans included a pharmacodynamic assay utilizing a murine model of fertility, we elected to assess PK parameters in female mice after IV dosing (5 mg/kg). **VU6032735** provided what could potentially be an ideal PK profile for a nonhormonal contraceptive. **VU6032735** displayed low observed clearance (8.6 mL/min/kg) with a long half-life (33.7 h) and a high volume of distribution (23.6 L/kg). Given the longevity of sperm within the female reproductive tract (viable for up to 5 days), a drug with a long half-life and a high volume of distribution may provide the ideal candidate for a nonhormonal female contraceptive.

Finally, utilizing **VU6032735** as a tool compound, we evaluated drug exposure and distribution in the various anatomical compartments of the murine FRT (ovary, oviduct, and uterus) after IP dosing. An extensive literature search provided no relevant information on possible protocols to accomplish this task. Given that the murine FRT is small as a whole and we would be dissecting out multiple regions of the FRT, we were concerned that a pool size of 1 animal would not provide us with sufficient sample for LC-MS/MS analysis. Therefore, in a systematic approach, we first set out to identify the appropriate sample/pool size. Samples were collected 30 min after dosing animals IP with pool sizes ranging from 1 to 3 animals. It was determined that an animal pool size of 2 or 3 provided more consistent results by our LC-MS/MS analysis. With this knowledge in hand, we next assessed tissue exposure over a 24-h period after 10 mg/kg IP dosing while utilizing a pool size of two animals per group. **VU6032735** was able to sustain high tissue exposure over time as the total drug concentration in all anatomical compartments of the FRT was >1 μM up to 24 h post dosing in female mice. Most importantly, **VU6032735** obtained high tissue exposure levels in the oviduct, where fertilization occurs with total drug concentrations ∼4.4- to 5.5-fold higher than the mSLO3 IC_50_ at the 24-h time point. While the compound concentration in the oviduct appears to be concentrating 5-fold more than in the uterus and 10-fold more than in the ovary, it cannot be ruled out that the observed higher compound concentration in the oviduct at the earlier time points may be due in part to the IP route of administration resulting in increased absorption of the compound through blood vessels in the peritoneal cavity. Future studies will investigate the impact of the route of administration on the absorption and concentration of this compound in the female reproductive tract and organs. Although the ultimate goal is to measure compound concentration in the murine oviducal fluid, we were unable to obtain sufficient quantities of fluid needed for analysis from the mouse oviduct. Further work will need to be done in larger animals, where oviducal fluid is abundant enough to allow accurate measurement of compound concentration.

Moreover, it is important to note that the effect of these SLO3 inhibitors is reversible in patch-clamp experiments as channel activity recovers after washout. Further optimization is needed in the future to decrease the off-rate and better control the duration of the action. This property will be particularly important when considering delivery strategies such as localized application in females (e.g., vaginal rings) or reversible contraception in males.

In conclusion, we have reported the first highly potent and subtype-selective SLO3 inhibitors (**VU6047606** and **VU6032735**). These analogs underwent an extensive *in vitro* and *in vivo* DMPK assessment, and we demonstrated that **VU6032735** can be used as a tool compound to study drug exposure and distribution in the murine FRT. To our knowledge, this is the first report in which a compound has been administered via a nonvaginal route with subsequent studies examining drug exposure and distribution throughout the various compartments of the FRT. This research provides groundwork for others in the field to study drug exposure and distribution within the female reproductive tract.

## Methods

### Chemicals

HTS hits were resynthesized, and all novel SLO3 inhibitors were designed and synthesized in Professor Craig Lindsley’s lab at Vanderbilt University. Compound purity was determined as ≥98% pure by liquid chromatography–mass spectrometry. All compounds that progressed into *in vivo* studies were further verified with high-resolution mass spectrometry and nuclear magnetic resonance (^1^H and^13^C). Synthetic routes and experimental details can be found in the Supporting Information.

### Human Cell Lines

HEK293 cells were transfected with the bicistronic vector pBUD-CE4.1 containing the genes of interest and a gene conferring antibiotic resistance. Cells were exposed to gradients of selection antibiotics, and optimal growth conditions for the stable lines were as follows: SLO3 and γ2 (SLO3-γ2) supplemented with 700 μg/mL Zeocin (Invitrogen); human SLO1 and β1 (SLO1-β1) with 1 mg/mL G418 (Sigma) and 3 μg/mL puromycin (Corning, Thermo Fisher); and human SLO1 and β4 (SLO1-β4) with 250 μg/mL Zeocin and 3 μg/mL puromycin. All cell lines were grown in Dulbecco’s Modified Eagle Medium (Gibco, Thermo Fisher) supplemented with 10% fetal bovine serum, 1% penicillin–streptomycin (Millipore Sigma), and selection antibiotics. To generate cells expressing SLO1 and γ2, stable SLO1-expressing HEK293 cells were transiently transfected with expression vectors for γ2 and green fluorescent protein (GFP) in the presence of lipofectamine (Invitrogen) for 5 h.

The cells were then washed and incubated in supplemented Dulbecco’s Modified Eagle Medium for 48 h before use. Patch-clamp electrophysiology was performed only on cells expressing GFP.

### SLO3 Patch-Clamp Electrophysiology

Currents in HEK293 cells were measured in the whole-cell patch-clamp configuration with an Axopatch 200B amplifier, digitized with a Digidata 1440A, and interfaced with Clampex 10.6.0.13 (Molecular Devices). Recording solutions were prepared as previously described.^30,31^ Growth medium was aspirated and replaced with external recording solution (135 mM NaMeSO_3_, 5 mM KMeSO_3_, 2 mM MgCl_2_, 10 mM HEPES, 10 mM MES, pH 7.2). An isolated cell was located on an Axiovert 200 microscope (Zeiss), and a gigaohm seal was formed with a borosilicate pipette with resistance between 3 and 6 MΩ and filled with internal solution (140 mM KMeSO_3_, 10 mM HEPES, 10 mM MES, 1 mM EGTA, pH 7). The seal was then broken by applying a negative pressure. Cells were kept at a holding potential of −60 mV for 50 ms, tested with a stepped voltage protocol from −80 to +150 mV in 10 mV increments for 100 ms, and then dropped to −40 mV for 50 ms with 2.5 s between sweeps. This protocol was used to record currents present with increasing concentration of our compound of interest or 3 μL/mL vehicle (DMSO) in the bath solution. Individual cells were tested with between 1 and 5 concentrations of the compound of interest or until the patch was lost. Currents were allowed to stabilize before the initial recording and after the perfusion solution was changed before recording.

### Human Sperm Preparation

Human sperm samples were obtained by masturbation from deidentified patients after 3 to 5 days of abstinence. The Washington University Fertility and Reproductive Medicine Center confirmed that they met normal semen parameters according to the World Health Organization (≥15 million sperm per mL, ≥40% total motility, ≥32% progressive motility).^33^ Samples were allowed to liquefy for 1 h at room temperature. Sperm were purified by the swim-up method as previously described.^31,34^ Briefly, within 2 h of production, sperm were allowed to swim up in noncommercial human tubular fluid medium (HTF; 98 mM NaCl, 4.7 mM KCl, 0.4 mM KH_2_PO_4_, 2 mM CaCl_2_, 0.2 mM MgSO_4_, 20 mM HEPES, 3 mM glucose, 21 mM lactic acid, 0.3 mM sodium pyruvate, pH adjusted to 7.4 with NaOH, 285 mmol/kg osmolarity measured with a VAPRO Vapor Pressure Osmometer 5600, ELITechGroup, Belgium) for 1 h at 37 °C without CO_2_.

### Human Sperm Hyperactivated Motility

Human sperm collected by swim-up were incubated in capacitating media (HTF + 5 mg/mL of BSA + 25 mM NaHCO_3_ for 2 h). After this incubation hyperactivated motility was measured by computer-assisted sperm analysis (CASA) as previously described.^34^ Briefly, 3 μL of sperm suspension was loaded in a 20-μm Leja standard count four-chamber slide. Motility was measured with a HTR-CEROS II v.1.7 (Hamilton-Thorne Research). Sperm were classified as hyperactivated if they met the following criteria: curvilinear velocity (VCL) > 150 μm/s, lateral head displacement (ALH) > 7.0 μm, and linearity coefficient (LIN) < 50%.^8^ For acute inhibition experiments, 10 μM of our compound of interest was added for 1 min prior to recording motility. For inhibitor incubation experiments, the compound of interest or DMSO was added during the 2 h incubation in capacitating media.

### Isolation of Epididymal Mouse Spermatozoa

Electrophysiology: C57Bl/6 sexually mature male mice (60–90 days old) were euthanized by inhalation of CO_2_ followed by cervical dislocation and sperm were isolated from the *corpus* epididymis by swim-out. Then the sperm suspension was collected and centrifuged (500×*g*, 6 min) twice to purify the sperm. HEPES-buffered saline (HS) was used for sperm swim-out and storage. It contained (in mM): 135 NaCl, 5 KCl, 1 MgSO_4_, 2 CaCl_2_, 20 HEPES, 5 glucose, 10 lactic acid, and 1 Na·pyruvate at pH 7.4. The sperm suspension was kept at room temperature (∼22 °C) for 4–6 h until recording. All animal husbandry and experimental procedures were approved by the Animal Studies Committee of the Washington University in St. Louis School of Medicine.

### Expression of Mouse SLO3 Channels in HEK293 Cells

HEK293 cells were plated at a density of ∼400,000 cells per 40 mm dish the day before transfection. Lipofectamine 3000 transfection reagent (catalog #L3000015; Thermo Fisher Scientific) was used to transfect HEK293 cells following manufacturer’s instructions. Per 40 mm dish, 2.25 μg of mouse SLO3 and its auxiliary subunit γ2 cDNAs (1:1) were added to cell layers that were 70–90% confluent. As a marker for transfection, cells were cotransfected with pmaxGFP, a CMV plasmid expressing green fluorescent protein (Amaxa Biosystems) at 0.2 μg per dish. After transfection, the cells were incubated at 37 °C with 5% CO_2_ for 2–4 d until recording.

### Whole-Cell Recording from Mouse Sperm and HEK293 Cells

Voltage-clamp recordings were performed using an Axopatch 200B amplifier (Molecular Devices). Recordings were filtered at 5 kHz with the internal filter of the amplifier and digitized at 50 kHz using a Digidata 1550B digitizer (Molecular Devices). Recording pipettes were pulled from borosilicate glass with tip resistances of 3–8 MΩ after filling with pipette solution containing (in mM): 140 KMES, 1 EGTA, 10 HEPES, pH 7.4 with KOH. Bath solutions contained (in mM): 135 NaMES, 5 KMES, 2 MgCl_2_, and 10 HEPES, pH 7.4 with NaOH. Experiments were performed at room temperature (∼22 °C). Whole-cell currents were evoked from a holding potential of −60 mV in steps from −80 to +100 mV in 10 mV increments. Test compounds were prepared daily from a 10 mM stock solution (in DMSO).

For mouse sperm recordings, the pipettes were fire-polished with Microforge MF-830 (Narishige, Japan) to reach tip resistances of 8–12 MΩ. The giga-ohm (GΩ) seals were formed between the glass micropipette and the sperm cytoplasmic droplet in HS solution. Break-in to whole-cell mode was achieved by applying short (1–5 ms) voltage pulses of 488–888 mV.

### SLO3 Patch Clamp Data Analysis

Data were analyzed with a pClamp 10.6 (Molecular Devices).^32^ **VU6047606** and **VU6032735** inhibition dose–response relationships were fit by the Hill equation in OriginPro 7.5 (OriginLab Corporation): $R=\frac{1}{1+(C/IC_{50}{)}^{n}}+I_{off}$ , in which *R* is fractional unblock, *C* is compound concentration, IC_50_ is the half inhibition concentration, *n* is the Hill coefficient, and *I*_off_ is an offset item for leak current. Error bars in the figures represent the SDs. Each data point is averaged from at least 5 measurements. Curve-fitting results are reported as the fitted values with standard error, which was estimated by OriginPro according to the Error Propagation formula.

### hERG Patch Clamp Electrophysiology

Whole-cell patch-clamp electrophysiology was used to record hERG currents from a stably transfected monoclonal cell line expressing human hERG. The HEK-293-hERG cell line was generously provided by Dr. David Weaver (Vanderbilt University). Cells were cultured in a 5% CO_2_ incubator at 37 °C, dissociated with 0.25% Trypsin-EDTA, and seeded onto 12 mm circular coverslips (Electron Microscopy Sciences, PA, USA) for same-day whole-cell patch-clamp recordings. hERG currents were recorded at room temperature by using an AxoPatch 200B amplifier with Clampex 10.7 software (Molecular Devices, USA). The voltage-clamp protocol consisted of a 4-s step to +30 mV from a −80 mV holding potential, followed by a 4-s step to −40 mV, repeated every 10 s. Data were sampled at 5 kHz and filtered at 1 kHz. Patch pipettes, pulled from 1.5 mm OD thin-walled capillaries (Warner Instruments, MA, USA) using a Sutter P-1000 puller (Sutter Instrument, CA, USA), had resistances of 2–3 MΩ when filled with intracellular solution containing (mM): 135 KCl, 10 HEPES, 1 EGTA, 2 MgCl_2_, 2 Na_2_ATP (pH 7.35, adjusted with KOH). The extracellular solution contained (mM): 135 NaCl, 5 KCl, 2 CaCl_2_, 1 MgCl_2_, 5 Glucose, 10 HEPES, and 10 Sucrose (pH 7.35, adjusted with NaOH). After establishing stable baseline hERG currents, cells were perfused with compounds at concentrations of 0.03–30 μM until response saturation (typically 2–8 min). Recordings concluded with a 10 μM Quinidine solution to isolate Quinidine-sensitive currents, with compound responses expressed as fractions of this current. Peak “tail” current amplitudes at the beginning of −40 mV steps were analyzed using Clampfit 11.2 (Molecular Devices). IC_50_ values were calculated by fitting the Hill equation to concentration–response curves (CRCs) using the built-in *Y* = Bottom + (Top – Bottom)/(1 + (IC_50_/*X*)^HillSlope^) model (nonlinear regression fit: [Inhibitor] vs response – Variable slope (four parameters)) in GraphPad Prism 10.4 (GraphPad Software).

### Plasma Protein Binding (Mouse, Rat, Human)

Determination of compounds’ fraction unbound (*f*_*u*_) in plasma from mouse, rat, and human was conducted *in vitro* via equilibrium dialysis using HTDialysis membrane plates. Dialysis membranes were hydrated as described by the manufacturer and inserted into the HTD plate, which was assembled and prepared for sample addition by the dispensing of blank buffer (Dulbecco’s Phosphate-Buffered Saline, 100 μL/well) into the *trans* chamber of each membrane-split well. Each compound was diluted into plasma from each species (5 μM final concentration), which was aliquoted in triplicate in the *cis* chamber of the prepared HTD plate wells. The HTD plate was sealed and incubated for 6 h at 37 °C. Following incubation, each well (both *cis* and *trans* chambers) was transferred (20 μL) to the corresponding wells of a 96-shallow-well (V-bottom) plate. The daughter plates were then matrix-matched (buffer-side wells received an equal volume of plasma, and plasma-side wells received an equal volume of buffer), and an extraction solution (120 μL; acetonitrile containing 50 nM carbamazepine as an internal standard (IS)) was added to all wells of both daughter plates to precipitate protein and extract the test article. The plates were then sealed and centrifuged (3500 rcf) for 10 min at ambient temperature. Supernatant (60 μL) from each well of the daughter plates was then transferred to the corresponding wells of new daughter plates (96-shallow-well, V-bottom) containing water (Milli-Q, 60 μL/well), and the plates were sealed in preparation for liquid chromatography–tandem mass spectrometry (LC-MS/MS) analysis (see below).

### Intrinsic Clearance in Mouse, Rat, and Human Liver Microsomes

The *in vitro* intrinsic clearance (CL_int_) was investigated in commercially obtained hepatic microsomes from mouse, rat, and human donors using the substrate depletion (i.e., loss-of-parent vs time, or half-life (*t*_1/2_) method) approach with analyte detection via liquid chromatography–tandem mass spectrometry (LC-MS/MS). For each species, mean %parent remaining values at each time point were calculated from replicate raw data (analyte:IS peak area ratios) and used to determine *in vitro t*_1/2_ and CL_int_.

Experiments were carried out by using a robot-assisted liquid handler (Bravo with BenchCell, Agilent). The compound was incubated (1 μM final concentration) in buffer (100 mM potassium phosphate pH 7.4 with 3 mM MgCl_2_) containing hepatic microsomes (0.5 mg/mL final concentration) from each species, discretely, at 37 °C under constant orbital shaking. After 5 min (preincubation), reactions were initiated by the addition of nicotinamide adenine dinucleotide phosphate (NADPH, 1 mM final concentration). At selected time intervals (0, 3, 7, 15, 25, and 45 min) postaddition of NADPH, aliquots (50 μL) were taken and placed into a 96-shallow-well plate containing ice-cold acetonitrile (150 μL) with carbamazepine (IS, 50 nM). The plates were then centrifuged (3000 rcf at 4 °C) for 10 min. The supernatants were transferred to a new 96-shallow-well daughter plate and diluted (1:1 v/v) with water (Milli-Q filtered). The plates were then sealed for preparation for LC-MS/MS analysis (see below).

Analyte:IS peak area ratios generated by LC-MS/MS were used to construct natural log-transformed %parent remaining vs time plots (using *t* = 0 min post-NADPH addition sample data as the starting point set to 100%). *In vitro* compound *t*_1/2_ values were calculated by using the following equation:

$$t_{1/2}=\frac{\ln\left(2\right)}{k}$$

where *k* is the slope from the linear regression analysis of the natural log-transformed data (using means from all replicates at each time point). Resulting *t*_1/2_ values were then used to calculate hepatic CL_int_ values according to the following equation and with the use of species-specific scale-up factors for liver weight (grams) per total body weight (kg):

CLint=0.693invitrot1/2×1mLincubation0.5mgmicrosomes×45mgmicrosomes1gramliver×garamliverkgbodywt

^a^Scale-up factors used are 45 (rat) and 20 (human).^35^

Predicted hepatic clearance (CL_hep_) was calculated by using the following equation:

$${CL}_{hep}=\frac{Q_{h}\times {CL}_{int}}{Q_{h}+{CL}_{int}}$$

*Q*_h_ represents hepatic blood flow (mL/min/kg): 21 for humans, 70 for rats, and 90 for mouse.

### Animals

Experimental animals were male Sprague–Dawley rats and female C57Bl/6 mice.

### Ethics Statement

All animal procedures used in this study adhered to the published guidelines of the National Institutes of Health and were approved by the Institutional Animal Care and Use Committee at Vanderbilt University Medical Center and Washington University. Animals were maintained in an AAALAC-accredited facility in 12 h light/dark cycles with food and water ad libitum.

### *In Vivo* Pharmacokinetics (Rat Cassette and Mouse Discrete)

*In-life phase*—Compounds were formulated in 12% ethanol, 48% PEG400, and 40% DMSO (v/v/v) at a concentration of 1 mg/mL and administered as a single 0.2 mg/kg IV dose (1 mL/kg) to male, Sprague–Dawley rats (*n* = 1; 342 g body weight) via injection into a surgically implanted jugular vein catheter. Blood samples were collected serially from a surgically implanted carotid artery catheter in each animal over multiple postadministration time points (0.033, 0.117, 0.25, 0.5, 1, 2, 4, 7, and 24 h) into chilled, K_2_EDTA anticoagulant-fortified tubes and immediately placed on wet ice. The blood samples were then centrifuged (1700 rcf, 5 min, 4 °C) in order to obtain plasma samples, which were stored at −80 °C until analysis by LC-MS/MS. **VU6032735** was also dosed IV in C57Bl6 mice at 5 mg/kg IV to determine mouse PK. Formulation and experimental design were identical to those used for rats except that whole blood was used for analysis.

Plasma samples from the in-life phase of the study were thawed at ambient temperature (benchtop), and then, aliquots (20 μL per sample) were transferred to a 96-shallow-well (V-bottom) plate. Matrix-matched quality control (QC) samples and a standard curve of the compound (1 mg/mL DMSO stock solution) were prepared in blank rat plasma (K_2_EDTA-treated) via serial dilution and transferred (20 μL each) to the plate along with multiple blank plasma and tissue homogenate samples. Acetonitrile (120 μL) containing IS (10 nM carbamazepine) was added to each well of the plate to precipitate the protein. The plate was then centrifuged (4000 rcf, 5 min, ambient temperature), and the resulting supernatants (60 μL each) were transferred to a new 96-shallow-well (V-bottom) plate containing an equal volume (60 μL per well) of water (Milli-Q purified). The plate was then sealed for LC-MS/MS analysis.

### LC-MS/MS Analysis

Samples were analyzed via electrospray ionization (ESI) LC-MS/MS on an AB Sciex 4500 (Foster City, CA) triple-quadrupole instrument that was coupled with an Agilent 1290 Infinity Binary Pump and multisampler (Santa Clara, CA) and concentrations (ng/mL or ng/g) were determined using a matrix-matched 10-point standard curve and then converted to nM concentrations. Mass spectrometer conditions are listed in Table 1. Quantitation of compounds was performed via AB Sciex Multiquant software using the raw analyte:IS peak area ratios. The typical detection range was 0.5 ng/mL to ≥5000 ng/mL utilizing a quadratic equation regression with 1/*x*² weighting. Correction for dilution of all tissue samples was performed postquantitation. The corrections for dilution in the extraction buffer employed correction factors specific to each tissue weight.^29^

### Multispecies Metabolite Identification

Metabolites were identified after preincubating each compound at a 10 μM final concentration with 2.5 × 10^5^ hepatocytes from human, dog, cynomolgus monkey, and rat in 200 μL of Opti_incubate_ buffer (BioIVT, Westbury, NY). Samples were quenched with equal volumes of acetonitrile containing an internal standard at 0 and 180 min. After centrifugation, 60 μL of the supernatant was transferred to 60 μL of water for LC-MS analysis. Samples were analyzed using a Thermo Scientific Vanquish UHPLC consisting of a VH-P10-A pump, a VH-A10-A sampler module, a VH-C10-A column compartment, and the VF-D11-A UV detector coupled to an Orbitrap Fusion Lumos instrument with UVPD. A binary solvent system consisting of (A) water with 0.1% formic acid and (B) acetonitrile with 0.1% formic acid was used with an Accucore Vanquish C18+, 2.1 mm × 100 mm, 1.5 μm, UHPLC column maintained at 40 °C. The data generated by data-dependent acquisition via normal acquisition and AcquireX were processed manually with Thermo Scientific FreeStyle and assisted by Compound Discoverer 3.3.1.111 to aid in the structural assignments.

### Off-Target Safety Screen

Both lead compounds, **VU6047606** and **VU6032735**, were subjected to radioligand binding assays in a Eurofins LeadProfilingScreen SafetyScreen Panel to detect potential adverse activity, relative selectivity, and off-target activity. Compounds were screened at 10 μM and percent inhibition was monitored across 68 potential off-target G protein-coupled receptors, ion channels, nuclear hormone receptors, and transporters.^29,36^

### Determining the Distribution Ratio between the Murine Female Reproductive Organs versus Plasma

*In-life phase*—Compounds were formulated in PEG400:DMSO (4:1, v/v) at a concentration of 1 mg/mL and administered as a single 1 mg/kg dose (1 mL/kg) to female C57Bl/6 mice (*n* = 2–4; 30 g body weight) via intravenous (i.v.) injection. Blood samples were collected serially from a surgically implanted carotid artery catheter in each animal over multiple postadministration time points (0.25, 0.5, 1, 3, 6, and 24 h) into chilled, K_2_EDTA anticoagulant-fortified tubes and immediately placed on wet ice. The blood samples were stored at −80 °C until analysis by LC-MS/MS.

At the same time points, female reproductive organs were obtained by rapid dissection, rinsed with PBS, pooled by lots of 2 and immediately frozen in individual tissue collection boxes (dry ice). All tissues and blood samples were stored at −80 °C until analysis by LC-MS/MS.

### Analysis of Drug Concentrations in Various Anatomical Compartments of the Murine FRT

In subsequent experiments, mice were dosed by injection into the peritoneum (ip) as a single 10 mg/kg dose, the reproductive organs were dissected, and the ovaries, the oviducts, and the uterus were pooled from 2 animals and frozen. Tissues were subjected to mechanical homogenization (Mini-BeadBeater, BioSpec Products, Inc., Bartlesville, OK) in the presence of stainless-steel beads (1.0 mm) and extraction buffer (isopropanol:water, 7:3, v/v; 1 mL per sample for whole reproductive organs and uterus and 250 μL for ovaries and oviducts). Homogenized tissue samples were then centrifuged (4000 rcf, 5 min, ambient temperature), and 5 μL of the supernatant was used for quantification of the analyte. The plasma standard curve and QCs were used for compound quantitation in reproductive tissues.

## Supplementary Material



## Data Availability

The authors declare that all data supporting the findings of this study are contained within the paper and its Supporting Information and/or available on request from the corresponding authors.

## Notes

General methods for the synthesis and characterization for key compounds, conditions used for DMPK LC-MS/MS analysis, and additional ancillary pharmacology data (Eurofins Panlabs LeadProfilingScreen, multispecies hepatocyte MetID) (PDF)

## Abbreviations

CYP: Cytochrome P450
DMSO: dimethyl sulfoxide
*f*_*u*_: fraction unbound
FRT: female reproductive tract
GFP: green fluorescent protein
hERG: human ether-a-go-go channel
HTS: high-throughput screen
CL_hep_: intrinsic clearance
CL_int_: intrinsic clearance
CL_p_: plasma clearance
IS: internal standard
IUDs: intrauterine devices
IVPK: intravenous pharmacokinetics
K^+^: potassium
KCNU1: Potassium Calcium-Activated Channel Subfamily U Member 1 gene
LARCs: long-acting reversible contraceptives
LC-MS/MS: liquid chromatography–tandem mass spectrometry
PK: pharmacokinetics
SLO3: the sperm-specific potassium channel
*t*_1/2_: half-life
*V*_ss_: volume of distribution
