# Supplementary material for: Highly Potent and Subtype-Selective Sperm-Specific Potassium Channel SLO3 Inhibitors Display High Tissue Exposure in the Murine Female Reproductive Tract
Source: ACS Pharmacol Transl Sci. 2025 Aug 15;8(9):3281–95. doi: 10.1021/acsptsci.5c00416 (PMC12441834; doi:10.1021/acsptsci.5c00416)
Supplement: Supplementary file 1 [file pt5c00416_si_001.pdf]

Highly Potent and Sub-Type Selective Sperm-Specific Potassium Channel 3 Inhibitors Display High Tissue Exposure in the Murine Female Reproductive Tract

Kayla J. Temple[a,b*†], Ping Li[h†], Hallie G. McKinnie[a,b], Analisa Thompson Gray[a,b], Jeanette L. Bertron[a,b], Anna E. Ringuette[a,b], Pedro de Andrade Horn[a,b], Maximilian D. Lyon[h], Shweta Bhagwat[h], Leila Asadi[h], Sophia Li[h], Roman M. Lazarenko[e], Ronald McCarthy[h], Sichen Chang[a,b], Jeremy A. Turkett[a,b], Valerie Kramlinger[a,b], Katherine J. Watson[a,b], Irene A. Zagol-Ikapitte[a,b], Jerod S. Denton[b,e,g], Celia M. Santi[h], Carrie K. Jones[a,b,f], Craig W. Lindsley[a,b,c,d], Olivier Boutaud[a,b*]

[a]Warren Center for Neuroscience Drug Discovery, Vanderbilt University, Nashville, TN 37232, USA
[b]Department of Pharmacology, Vanderbilt University School of Medicine, Nashville, TN 37232, USA
[c]Department of Chemistry, Vanderbilt University, Nashville, TN 37232, USA
[d]Department of Biochemistry, Vanderbilt University, Nashville, TN 37232, USA
[e]Department of Anesthesiology, Vanderbilt University Medical Center, Nashville, TN 37232
[f]Vanderbilt Brain Institute, Vanderbilt University School of Medicine, Nashville, TN 37232, USA
[g]Vanderbilt Institute of Chemical Biology, Vanderbilt University, Nashville, TN 37232
[h]Department of Obstetrics and Gynecology, Washington University School of Medicine, St. Louis, MO 63110

[†]Authors contributed equally
*Corresponding authors' email:
kayla.temple@vanderbilt.edu.
olivier.boutaud@vanderbilt.edu

Table of Contents

Experimental Synthetic Procedures and Spectroscopic Data S2
General Synthetic Methods. S2
General Instrumentation Methods. S2
Synthesis of Key Compounds. S4
Conditions Used for LC-MC/MS Analysis S16
Ancillary Pharmacology S17
Lead Profiling Screen – Eurofins Panlabs for **VU6032735** S17
Lead Profiling Screen – Eurofins Panlabs for **VU6047606** S19
Multi-species hepatocyte MetID S21
Inhibition of human SLO3-γ2 currents in HEK293 cells S23
Inhibition of mouse SLO3-γ2 currents in HEK293 cells S24
Inhibition of KSper currents in mouse sperm S25

Experimental Synthetic Procedures and Spectroscopic Data

General Synthetic Methods.

All reactions were carried out employing standard chemical techniques. Solvents used for extraction, washing, and chromatography were HPLC grade. All reagents were purchased from commercial sources and were used without further purification. Chiral *tert*-butyl 3-fluoro-4-hydroxypiperidine-1-carboxylate*s* (**9a-d**) were obtained commercially from the following vendors: *tert*-butyl (3*R*,4*S*)-3-fluoro-4-hydroxypiperidine-1-carboxylate (**9a**), 97% purity – Synthonix (CAS 1174020-42-8); *tert*-butyl (3*R*,4*R*)-3-fluoro-4-hydroxypiperidine-1-carboxylate (**9b**), 97% purity – Pharmablock (CAS 1174020-43-9); *tert*-butyl (3*S*,4*R*)-3-fluoro-4-hydroxypiperidine-1-carboxylate (**9c**), 97% purity – Synthonix (CAS 1174020-40-6); *tert*-butyl (3*S*,4*S*)-3-fluoro-4-hydroxypiperidine-1-carboxylate (**9d**), 97% purity – Pharmablock (CAS 1174020-44-0).

Automated flash column chromatography was performed on a Biotage Isolera 1 or a Teledyne ISCO CombiFlash system. RP-HPLC was performed on a Gilson preparative reversed-phase HPLC system comprised of a 333 aqueous pump with solvent-selection valve, 334 organic pump, GX-271 or GX-281 liquid hander, two column switching valves, and a 155 UV detector. Absorbance was typically monitored at 215 or 220 nm. Column: Phenomenex Axia-packed Gemini C18, 5 µm. Mobile phase: CH_3CN in H_2O (0.1% TFA) or CH_3CN in H_2O (0.05% *v/v* NH_4OH) under the specified gradient, then hold 95% CH_3CN in 5% aqueous phase, 50 mL/min, 23° C. All compounds were found to be >95% pure by LCMS analysis.

Safety statement*: no unexpected or unusually high safety hazards were encountered.*

General Instrumentation Methods.

All NMR spectra were recorded on a 400 MHz AMX Bruker NMR spectrometer. ^1H and ^{13}C chemical shifts are reported in δ values in ppm downfield with the deuterated solvent as the internal

standard. Data are reported as follows: chemical shift, multiplicity (s = singlet, d = doublet, t = triplet, q = quartet, b = broad, m = multiplet), integration, coupling constant (Hz).

Low resolution mass spectra (LRMS) were obtained on an Agilent 6120/6150 or Waters QDa (Performance) SQ MS with ESI source. *Method A (Agilent 6120/6150):* MS parameters were as follows: fragmentor: 70, capillary voltage: 3000 V, nebulizer pressure: 30 psig, drying gas flow: 13 L/min, drying gas temperature: 350 ºC. Samples were introduced via an Agilent 1290 UHPLC comprised of a G4220A binary pump, G4226A ALS, G1316C TCC, and G4212A DAD with ULD flow cell. UV absorption was generally observed at 215 nm and 254 nm with a 4 nm bandwidth. Column: Waters Acquity BEH C18, 1.0 x 50 mm, 1.7 um. Gradient conditions: 5% to 95% CH_3CN in H_2O (0.1% TFA) over 1.4 min, hold at 95% CH_3CN for 0.1 min, 0.5 mL/min, 55 ºC. *Method B (Agilent 6120/6150):* MS parameters were as follows: fragmentor: 100, capillary voltage: 3000 V, nebulizer pressure: 40 psig, drying gas flow: 11 L/min, drying gas temperature: 350 ºC. Samples were introduced via an Agilent 1200 HPLC comprised of a degasser, G1312A binary pump, G1367B HP-ALS, G1316A TCC, G1315D DAD, and a Varian 380 ELSD (if applicable). UV absorption was generally observed at 215 nm and 254 nm with a 4 nm bandwidth. Column: Thermo Accucore C18, 2.1 x 30 mm, 2.6 um. Gradient conditions: 7% to 95% CH_3CN in H_2O (0.1% TFA) over 1.6 min, hold at 95% CH_3CN for 0.35 min, 1.5 mL/min, 45 ºC. *Method C (Waters QDa (Performance) SQ MS)*: MS parameters were as follows: cone voltage: 15 V, capillary voltage: 0.8 kV, probe temperature: 600° C. Samples were introduced via an Acquity I-Class PLUS UPLC comprised of a BSM, FL-SM, CH-A, and PDA. UV absorption was generally observed at 215 nm and 254 nm; 4 nm bandwidth. Column: Phenomenex EVO C18, 1.0 x 50 mm, 1.7 um. Column temperature: 55° C. Flow rate: 0.4 mL/min. Default gradient: 5% to 95% CH_3CN (0.05% TFA) in H_2O (0.05% TFA) over 1.4 min (curve 6), hold at 95% CH_3CN for 0.1 min. "Polar" (2% to 70% CH_3CN (0.05% TFA) in H_2O (0.05% TFA) over 0.8 min (curve 6), transition to 95% CH_3CN over 0.1 min (curve 6), hold at 95% CH_3CN for 0.6 min.) and "Non-Polar" (40% to 95% CH_3CN (0.05% TFA) in H_2O (0.05% TFA) over 1.4 min (curve 6), hold at 95% CH_3CN for 0.1 min.) gradients were also available. *Method D (Waters QDa (Performance) SQ MS):* MS parameters were as follows: cone voltage: 15 V, capillary voltage: 0.8 kV, probe temperature: 600° C. Samples

were introduced via an Acquity I-Class PLUS UPLC comprised of a BSM, FL-SM, CH-A, and PDA. UV absorption was generally observed at 215 nm and 254 nm with a 4 nm bandwidth. Column: Phenomenex EVO C18, 1.0 x 50 mm, 1.7 um. Column temperature: 55° C. Flow rate: 0.4 mL/min. Default gradient: 5% to 95% CH_3CN in H_2O (5 mM NH_4HCO_3) over 1.4 min (curve 6), hold at 95% CH_3CN for 0.1 min. "Polar" (2% to 70% CH_3CN in H_2O (5 mM NH_4HCO_3) over 0.8 min (curve 6), transition to 95% CH_3CN over 0.1 min (curve 6), hold at 95% CH_3CN for 0.6 min.) and "Non-Polar" (40% to 95% CH_3CN in H_2O (5 mM NH_4HCO_3) over 1.4 min (curve 6), hold at 95% CH_3CN for 0.1 min.) gradients were also available.

High resolution mass spectra (HRMS) were obtained on an Agilent 6540 UHD Q-TOF with ESI source. MS parameters were as follows: fragmentor: 150, capillary voltage: 3500 V, nebulizer pressure: 60 psig, drying gas flow: 13 L/min, drying gas temperature: 275 ºC. Samples were introduced via an Agilent 1200 UHPLC comprised of a G4220A binary pump, G4226A 3 ALS, G1316C TCC, and G4212A DAD with ULD flow cell. UV absorption was observed at 215 nm and 254 nm with a 4 nm bandwidth. Column: Agilent Zorbax Extend C18, 1.8 μm, 2.1 x 50 mm. Gradient conditions: 5% to 95% CH_3CN in H_2O (0.1% formic acid) over 1 min, hold at 95% CH_3CN for 0.1 min, 0.5 mL/min, 40 ºC.

Synthesis of Key Compounds.

Scheme 1. Synthesis of SLO3 inhibitor **VU6032735**.[a]

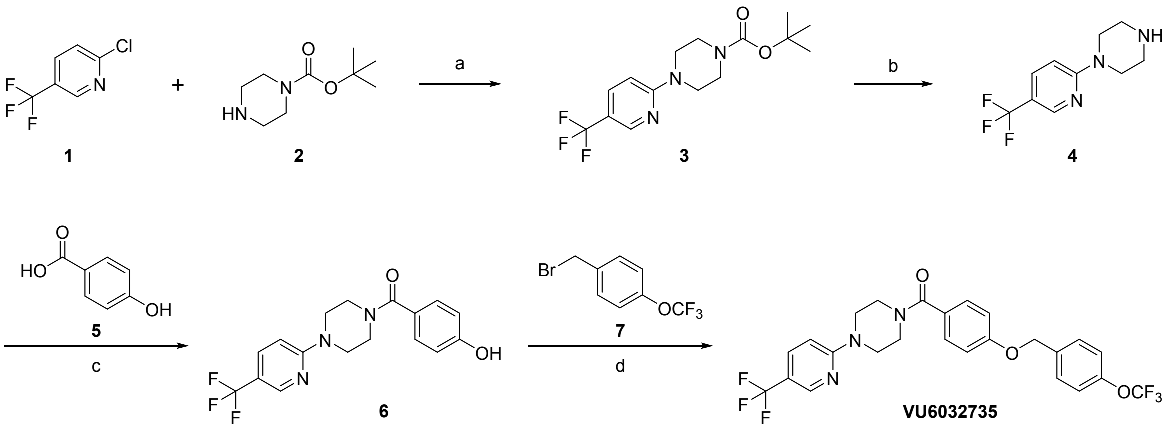

[a] Reagents and conditions: (a) K_2CO_3, DMF, 90 °C, 2h, 81%; (b) TFA, DCM, 0.5h, 94%; (c) **5**, DIEA, HATU, DCM, 40 °C, 18h, 96%; (d) **7**, K_2CO_3, DMF, 50 °C, 22h, 68%.

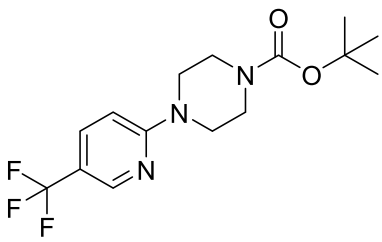

***tert*-Butyl 4-[5-(trifluoromethyl)pyridin-2-yl]piperazine-1-carboxylate (3)**. To a 250 mL round bottom flask charged with a stir bar was added *tert*-butyl piperazine-1-carboxylate (**2**, 10.0 g, 53.7 mmol), 2-chloro-5-(trifluoromethyl)pyridine (**1**, 11.7 g, 8.3 mL, 64.4 mmol), K_2CO_3 (15.1 g, 107.4 mmol), and DMF (67.1 mL). The reaction mixture was stirred at 90 °C for 18 hours. After cooling to ambient temperature, saturated aqueous NH_4Cl (50 mL) was added and the mixture was extracted with EtOAc (50 mL). After sequential washing with water (3x) and brine, the organics were dried ($MgSO_4$), filtered, and concentrated under reduced pressure. The crude material was purified using normal-phase flash chromatography on silica gel (5-25% EtOAc/hexanes) to afford 14.3 g of title compound (81% yield). LRMS: $C_{15}H_{20}F_3N_3O_2$ $[M+H]^+$ calc. mass 332.1, found 332.2.

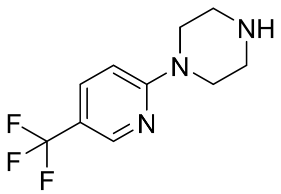

1-[5-(Trifluoromethyl)pyridin-2-yl]piperazine (4). To a round bottom flask charged with a stir bar, was dissolved tert-butyl 4-(5-(trifluoromethyl)pyridin-2-yl)piperazine-1-carboxylate (**3**, 7.0 g, 21.1 mmol) in DCM (28 mL). Next, trifluoroacetic acid (30.7 mL, 401.4 mmol) was added slowly at room temperature. After complete addition, the reaction mixture was allowed to stir at ambient temperature for 30 minutes then the reaction mixture was concentrated *in vacuo*. The crude residue was redissolved in DCM and sequentially washed with saturated aqueous $NaHCO_3$ solution (3x) and brine. The organic layer was dried ($MgSO_4$), filtered, and concentrated under reduced pressure to afford 4.6 g of title compound which was

carried forward without further purification (94% yield). LRMS: $C_{10}H_{12}F_3N_3$ $[M+H]^+$ calc. mass 232.1, found 232.2

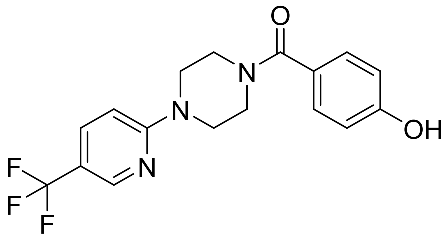

(4-Hydroxyphenyl)-[4-[5-(trifluoromethyl)pyridin-2-yl]piperazin-1-yl]methanone (6). To a solution of 1-(5-(trifluoromethyl)pyridin-2-yl)piperazine (**4**, 4.6 g, 20.1 mmol), 4-hydroxybenzoic acid (**5**, 2.8 g, 20.1 mmol), and HATU (8.4 g, 22.1 mmol) in DCM (50 mL) was added DIEA (5.2 mL, 30.1 mmol) at room temperature. The reaction mixture was then heated to 40 °C for 18 hours. After cooling to ambient temperature, water (50 mL) was added to the reaction mixture and the organic layer was isolated. The aqueous layer was then further extracted with DCM (3 x 50 mL). The combined organics were washed with brine, dried ($MgSO_4$), filtered, and concentrated *in vacuo*. The crude residue was purified using normal-phase flash chromatography on silica gel (40-100% EtOAc/hexanes) to afford 6.8 g of title compound (96% yield). LRMS: $C_{17}H_{16}F_3N_3O_2$ $[M+H]^+$ calc. mass 352.1, found 352.2.

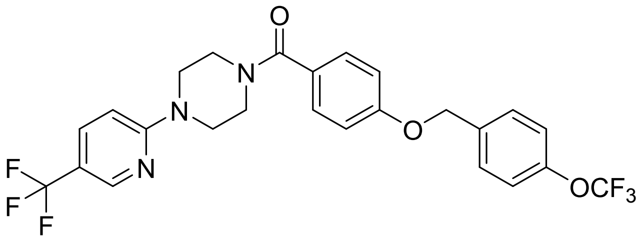

[4-[[4-(Trifluoromethoxy)phenyl]methoxy]phenyl]-[4-[5-(trifluoromethyl)pyridin-2-yl]piperazin-1-yl]methanone (VU6032735). To a solution of (**6**, 4-hydroxyphenyl)(4-(5-(trifluoromethyl)pyridin-2-yl)piperazin-1-yl)methanone (6.0 g, 17.1 mmol) and K_2CO_3 (7.2 g, 51.2 mmol) in DMF (11.4 mL) was added 1-(bromomethyl)-4-(trifluoromethoxy)benzene (**7**, 6.8 mL, 42.7 mmol) at room temperature. Upon complete addition, the reaction mixture heated at 50 °C for 22 hours. After cooling to ambient temperature,

the reaction mixture was diluted with DCM (20 mL) and washed sequentially with water (20 mL, 5x) and brine. The organic layer was then dried ($MgSO_4$), filtered, and concentrated under reduced pressure. The crude residue was purified using normal-phase flash chromatography on silica gel (40% EtOAc/hexanes) to afford 6.13 g of title compound as a white solid (68% yield). ^{1}H NMR (400 MHz, DMSO-d_6) δ 8.46 – 8.40 (m, 1H), 7.83 (dd, J = 9.2, 2.6 Hz, 1H), 7.65 – 7.57 (m, 2H), 7.48 – 7.37 (m, 4H), 7.13 – 7.05 (m, 2H), 6.96 (d, J = 9.1 Hz, 1H), 5.20 (s, 2H), 3.70 (s, 4H), 3.60 (s, 4H). ^{13}C NMR (101 MHz, DMSO-d_6) δ 169.1, 160.0, 159.2, 147.9, 145.3 (q), 136.3, 134.6 (q), 129.7 (2C), 129.2 (2C), 128.0, 124.8 (d), 121.1 (2C), 120.1 (d), 114.5 (2C), 113.5 (q), 106.4, 68.4, 44.1 (4C). HRMS (TOF, ESI) calc'd for $C_{25}H_{21}F_6N_3O_3$ $[M+H]^+$ = 526.1560, found = 526.1556.

Scheme 2. Representative synthesis of SLO3 inhibitors **VU6047606**, **VU6047607**, **VU6047608**, and **VU6047609**.[a]

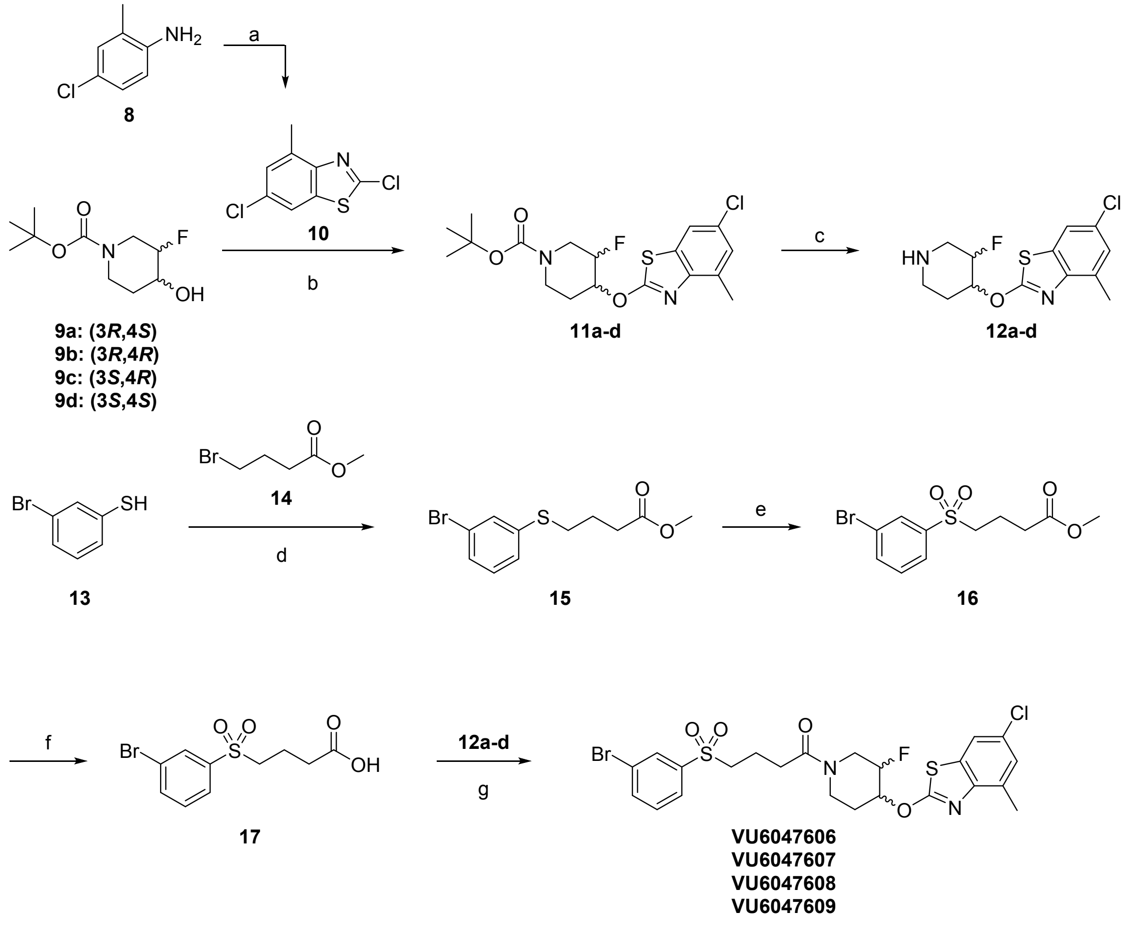

[a] Reagents and conditions: (a) i. NH_4SN, $BTMABr_3$, MeCN, 90 °C, 18h; ii. $CuCl_2$, $^tBuNO_2$, MeCN, 40 °C, 2h, 60% over 2 steps; (b) **10**, NaH, DMF, 0 °C to room temperature, 18h, 48-68%; (c) TFA, DCM, 1h, 86-99%; (d) **14**, NaH, DMF, 0 °C to room temperature, 18h, 88%; (e) *m*-CPBA, DCM, 0 °C to room temperature, 4h, 79%; (f) NaOH, THF:MeOH (3:1), 1h, 76%; (g) **12a-d**, HATU, DIEA, DCM, 40 °C, 5h, 44-87%.

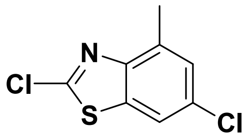

2,6-Dichloro-4-methyl-1,3-benzothiazole (10). Step 1: To a solution of 4-chloro-2-methylaniline (**8**, 4.0 g, 3.4 mL) in CH_3CN (115 mL) was added ammonium thiocyanate (4.1 g, 53.67 mmol), and the resulting reaction mixture was stirred at room temperature for 10 min. To this mixture was added dropwise a solution of benzyltrimethylammonium tribromide (14.3 g, 36.7 mmol) in CH_3CN (40 mL). The resulting solution was stirred at room temperature for 18 hours. Next, a saturated aqueous $NaHCO_3$ solution (20 mL) was added and the reaction mixture was extracted with EtOAc (3 × 50 mL). The combined organic layers were washed with brine, dried ($MgSO_4$), filtered, and concentrated *in vacuo* to afford 6-chloro-4-methylbenzo[*d*]thiazol-2-amine which was used directly in the next step without further purification. LRMS: $C_8H_7ClN_2S$ $[M+H]^+$ calc. mass 199.0, found 199.0.

Step 2: To a solution of $CuCl_2$ (2.3 g, 17.0 mmol) in CH_3CN (121 mL) was added tert-butyl nitrite (2.4 mL, 20.5 mmol) dropwise at 40 °C and the mixture was allowed to stir for 10 minutes. Next, a solution of 6-chloro-4-methyl-1,3-benzothiazol-2-amine (2.8 g, 14.1 mmol) in CH_3CN (96 mL) was added dropwise over 10 minutes. The reaction mixture was then allowed to stir for 2 hours at 40 °C. After cooling to ambient temperature, a 2*N* HCl solution (50 mL) was added, and the mixture was extracted with EtOAc (3 × 30 mL). The combined organic layers were washed sequentially with a saturated aqueous $NaHCO_3$ solution (20 mL) and brine then dried ($MgSO_4$), filtered, and concentrated *in vacuo*. The crude residue was purified

using normal-phase flash chromatography on silica gel (5% EtOAc/hexanes) to afford 1.9 g of title compound (60% yield). LRMS: $C_8H_5Cl_2NS$ $[M+H]^+$ calc. mass 218.0, found 217.9.

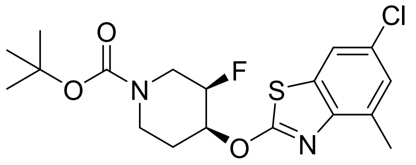

***tert*-Butyl (3*R*,4*S*)-4-[(6-chloro-4-methyl-1,3-benzothiazol-2-yl)oxy]-3-fluoropiperidine-1-carboxylate (11a).** A solution of 2,6-dichloro-4-methyl-1,3-benzothiazole (**10**, 100.mg, 0.46mmol) and *tert*-butyl (3*R*,4*S*)-3-fluoro-4-hydroxypiperidine-1-carboxylate (**9a**, 121 mg, 0.55 mmol) in DMF (4.6 mL) was cooled to 0 °C. Next, NaH (60% in mineral oil) (22 mg, 0.55 mmol) was added and the reaction was stirred at 0 °C for 10 minutes then warmed to room temperature for 18 hours. After cooling to 0 °C, water was added and the reaction mixture was extracted with EtOAc (3x). The combined organics were dried ($MgSO_4$), filtered, and concentrated *in vacuo*. The crude residue was purified using normal-phase flash chromatography on silica gel (0-50% EtOAc/hexanes) to afford 88.7 mg of title compound (48% yield). LRMS: $C_{18}H_{22}ClFN_2O_3S$ $[M+H]^+$ calc. mass 401.1, found 401.2.

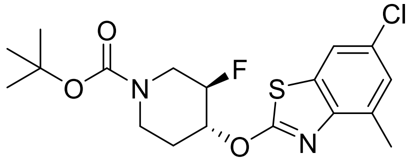

***tert*-Butyl (3*R*,4*R*)-4-[(6-chloro-4-methyl-1,3-benzothiazol-2-yl)oxy]-3-fluoropiperidine-1-carboxylate (11b).** Prepared in a similar manner as compound **11a** using 2,6-dichloro-4-methyl-1,3-benzothiazole (100 mg, 0.46 mmol), *tert*-butyl (3*R*,4*R*)-3-fluoro-4-hydroxypiperidine-1-carboxylate (121 mg, 0.55 mmol), NaH (60% in mineral oil) (22 mg, 0.55 mmol) and DMF (4.6 mL) to afford 124 mg of title compound (68% yield). LRMS: $C_{18}H_{22}ClFN_2O_3S$ $[M+H]^+$ calc. mass 401.1, found 345.1 (observed loss of *tert*-butyl group).

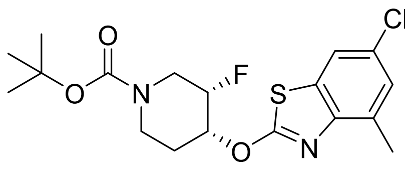

***tert*-Butyl (3*S*,4*R*)-4-[(6-chloro-4-methyl-1,3-benzothiazol-2-yl)oxy]-3-fluoropiperidine-1-carboxylate (11c).** Prepared in a similar manner as compound **11a** using 2,6-dichloro-4-methyl-1,3-benzothiazole (100 mg, 0.46 mmol), *tert*-butyl (3*S*,4*R*)-3-fluoro-4-hydroxypiperidine-1-carboxylate (121 mg, 0.55 mmol), NaH (60% in mineral oil) (22 mg, 0.55 mmol) and DMF (4.6 mL) to afford 123 mg of title compound (67% yield). LRMS: $C_{18}H_{22}ClFN_2O_3S$ $[M+H]^+$ calc. mass 401.1, found 345.1 (observed loss of *tert*-butyl group).

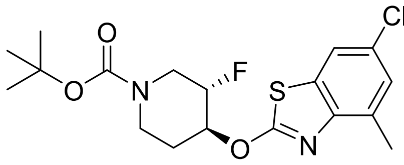

***tert-B*utyl (3*S*,4*S*)-4-[(6-chloro-4-methyl-1,3-benzothiazol-2-yl)oxy]-3-fluoropiperidine-1-carboxylate (11d).** Prepared in a similar manner as compound **11a** using 2,6-dichloro-4-methyl-1,3-benzothiazole (100 mg, 0.46 mmol), *tert*-butyl (3*S*,4*S*)-3-fluoro-4-hydroxypiperidine-1-carboxylate (121 mg, 0.55 mmol), NaH (60% in mineral oil) (22 mg, 0.55 mmol) and DMF (4.6 mL) to afford 114 mg of title compound (62% yield). LRMS: $C_{18}H_{22}ClFN_2O_3S$ $[M+H]^+$ calc. mass 401.1, found 345.1 (observed loss of *tert*-butyl group).

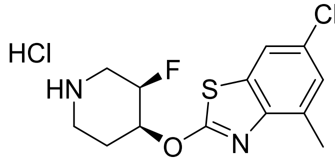

6-Chloro-4-methyl-2-[(3*R*,4*S*)-3-fluoropiperidin-4-yl]oxy-1,3-benzothiazole hydrochloride (12a). To a solution of *tert*-butyl (3*R*,4*S*)-4-[(6-chloro-4-methyl-1,3-benzothiazol-2-yl)oxy]-3-fluoropiperidine-1-carboxylate (2.14 g, 5.34 mmol) dissolved in 1,4-dioxane (5.0 mL) was added a 4*M* hydrochloric acid solution in 1,4-dioxane (8.0 mL, 32 mmol) at room temperature. The reaction was allowed to stir overnight

then the excess HCl and solvent were removed under reduced pressure to afford 1.73 g of title compound (80% yield) which was carried forward without further purification. LRMS: $C_{13}H_{14}ClFN_2OS$ $[M+H]^+$ calc. mass 301.1, found 301.2.

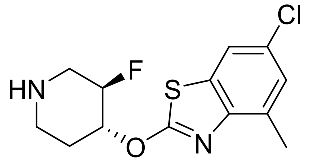

6-Chloro-4-methyl-2-[(3*R*,4*R*)-3-fluoropiperidin-4-yl]oxy-1,3-benzothiazole (12b). To a solution of *tert*-Butyl (3*R*,4*R*)-4-[(6-chloro-4-methyl-1,3-benzothiazol-2-yl)oxy]-3-fluoropiperidine-1-carboxylate (124 mg, 0.31 mmol) dissolved in DCM (1.3 mL) was slowly added trifluoroacetic acid (240 µL, 3.1 mmol) at room temperature. The reaction was allowed to stir for 1 hour after which excess TFA was removed under reduced pressure. The crude residue was taken up in DCM and sequentially washed with saturated aqueous $NaHCO_3$ solution (3x) and brine. The organic layer was dried ($MgSO_4$), filtered and concentrated to afford 86.4 mg of title compound (91% yield). LRMS: $C_{13}H_{14}ClFN_2OS$ $[M+H]^+$ calc. mass 301.1, found 301.2.

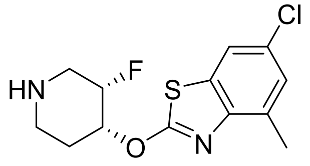

6-Chloro-4-methyl-2-[(3*S*,4*R*)-3-fluoropiperidin-4-yl]oxy-1,3-benzothiazole (12c). Prepared in a similar manner as compound **12b** using TFA (235 µL, 3.06 mmol), *tert*-butyl (3*S*,4*R*)-4-[(6-chloro-4-methyl-1,3-benzothiazol-2-yl)oxy]-3-fluoropiperidine-1-carboxylate (123 mg, 0.31 mmol) and DCM (1.2 mL) to afford 80.9 mg of title compound (86% yield). LRMS: $C_{13}H_{14}ClFN_2OS$ $[M+H]^+$ calc. mass 301.1, found 301.2.

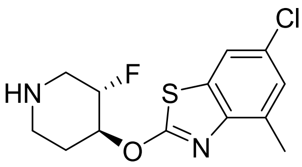

6-Chloro-4-methyl-2-[(3*S*,4*S*)-3-fluoropiperidin-4-yl]oxy-1,3-benzothiazole (12d). Prepared in a similar manner as compound **12b** using TFA (218 µL, 2.85 mmol), *tert*-butyl (3*S*,4*S*)-4-[(6-chloro-4-methyl-1,3-benzothiazol-2-yl)oxy]-3-fluoropiperidine-1-carboxylate (114 mg, 0.28 mmol) and DCM (1.1 mL) to afford 85.6 mg of title compound (98% yield). LRMS: $C_{13}H_{14}ClFN_2OS$ $[M+H]^+$ calc. mass 301.1, found 301.2.

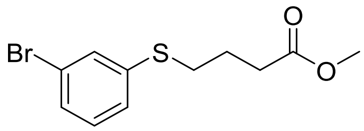

Methyl 4-((3-bromophenyl)thio)butanoate (15). A solution of 3-bromobenzenethiol (**13**, 4.37 mL, 42.3 mmol) in DMF (80 mL) was cooled to 0 °C where NaH (60% in mineral oil) (2.03 g, 50.8 mmol) was added and the mixture was allowed to stir for 10 minutes. Next, methyl 4-bromobutanoate (**14**, 6.41 mL, 50.8 mmol) was added, the cooling bath was removed, and the mixture was stirred overnight. The reaction mixture was cooled to 0 °C then diluted with water (60 mL) and extracted with EtOAc (50 mL, 3x). The combined organic layers were washed sequentially with with water (100 mL, 2x) and brine (100 mL) then dried ($MgSO_4$), filtered, and concentrated *in vacuo* to afford 10.75 g of title compound which was used directly in the next step (88% yield). LRMS: $C_{11}H_{13}BrO_2S$ $[M+H]^+$ calc. mass 289.0/291.0, found 289.2/291.2.

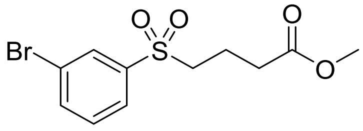

Methyl 4-(3-bromophenyl)sulfonylbutanoate (16). To a solution of methyl 4-((3-bromophenyl)thio)butanoate (**15**, 3.37 g, 11.7 mmol) dissolved in anhydrous DCM (14.5 mL) was added

3-chloroperoxybenzoic acid (5.75 g, 25.6 mmol) and the resulting mixture was stirred for 4 hours at room temperature. The reaction mixture was then basified with 2*N* NaOH (5 mL) and extracted with EtOAc (3 x 20 mL). The combined organic extracts were dried ($MgSO_4$), filtered, and concentrated *in vacuo*. The crude residue was purified using normal-phase flash chromatography on silica gel (5-80% EtOAc/hexanes) to afford 2.97 g of title compound (79% yield). LRMS: $C_{11}H_{13}BrO_4S$ $[M+H]^+$ calc. mass 321.0/323.0, found 321.2/323.2.

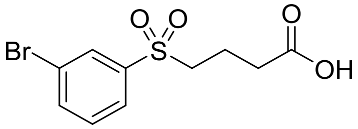

4-((3-Bromophenyl)sulfonyl)butanoic acid (17). To a solution of methyl 4-(3-bromophenyl)sulfonylbutanoate (**16**, 2.97 g, 9.2 mmol) dissolved in THF (5.8 mL) and methanol (18.5 mL) was added a 2*N* aqueous NaOH solution (18.5 mL, 36.95 mmol). The reaction mixture was stirred for 1 hour at that temperature. At this time, the pH was adjusted to a pH < 3 and the aqueous phase was extracted with EtOAc (3 x 5 ml). The combined organic layers were dried ($MgSO_4$), filtered, and concentrated *in vacuo* to afford 2.16 g of title compound (76% yield). LRMS: $C_{10}H_{11}BrO_4S$ $[M+H]^+$ calc. mass 307.0/309.0, found 307.0/309.0.

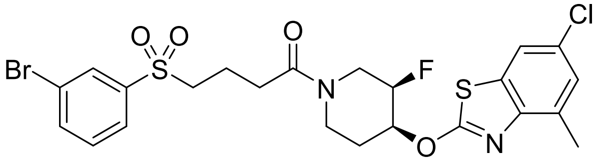

4-((3-Bromophenyl)sulfonyl)-1-((3*R*,4*S*)-4-((6-chloro-4-methylbenzo[*d*]thiazol-2-yl)oxy)-3-fluoropiperidin-1-yl)butan-1-one (VU6047606). To a solution of 4-(3-bromophenyl)sulfonylbutanoic acid (**17**, 50 mg, 0.16 mmol), 6-chloro-4-methyl-2-[(3*R*,4*S*)-3-fluoropiperidin-4-yl]oxy-1,3-benzothiazole (**12a**, 49 mg, 0.16 mmol), and HATU (68.1 mg, 0.18 mmol) in DCM (0.5 mL) was added DIEA (42.5 µL, 0.24 mmol). The reaction mixture was allowed to stir at 40 °C for 5 hours after which the reaction mixture was cooled to ambient temperature and concentrated *in vacuo.* The crude residue was purified using normal-

phase flash chromatography on silica gel (10-80% EtOAc/hexanes). Fractions containing desire product were concentrated and repurified using RP-HPLC (38-78% MeCN in 0.05% aqueous NH_4OH) to afford 47.1 mg of title compound (49% yield). ^{1}H NMR (400 MHz, CD_3CN) δ 8.04 (q, *J* = 1.5 Hz, 1H), 7.92 – 7.84 (m, 2H), 7.61 (d, *J* = 2.1 Hz, 1H), 7.55 (t, *J* = 7.9 Hz, 1H), 7.25 (dd, *J* = 2.0, 0.8 Hz, 1H), 5.50 – 5.32 (m, 1H), 5.26 – 4.99 (m, 1H), 4.60 – 3.96 (m, 1H), 4.41 – 3.72 (m, 1H), 3.51 – 2.87 (m, 4H), 2.53 (s, 3H), 2.47 (m, 2H), 2.08 (ddt, *J* = 12.5, 8.3, 4.0 Hz, 2H), 1.92 – 1.83 (m, 2H). ^{13}C NMR (CD_3CN, 101 MHz) δ 171.9 (d), 171.5 (d), 147.9, 142.2, 137.8, 133.7, 133.4, 132.3, 131.6, 129.3, 128.0, 127.9, 123.7, 119.6, 86.9 (d), 79.1 (dd), 55.6, 45.9 (dd), 41.4 (d), 31.3 (d), 26.7 (dd,), 19.5, 18.0. HRMS (TOF, ESI) calc'd for $C_{23}H_{23}BrClFN_2O_4S_2$ $[M+H]^+$ = 589.0028, found = 589.0031.

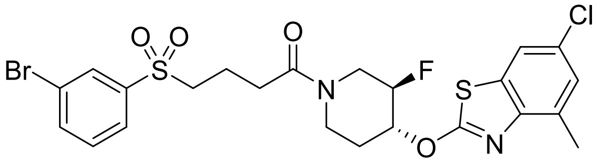

4-((3-Bromophenyl)sulfonyl)-1-((3*R*,4*R*)-4-((6-chloro-4-methylbenzo[*d*]thiazol-2-yl)oxy)-3-fluoropiperidin-1-yl)butan-1-one (VU6047607). Prepared in a similar manner as **VU6047606** using 4-(3-bromophenyl)sulfonylbutanoic acid (**17**, 50 mg, 0.16 mmol), 6-chloro-4-methyl-2-[(3*R*,4*R*)-3-fluoropiperidin-4-yl]oxy-1,3-benzothiazole (**12b**, 49 mg, 0.16 mmol), HATU (68.1 mg, 0.18 mmol), DIEA (42.5 µL, 0.24 mmol) and DCM (0.5 mL) to afford 83.5 mg of title compound (87% yield). ^{1}H NMR (400 MHz, CD_3CN) δ 8.04 (t, *J* = 1.9 Hz, 1H), 7.90 – 7.88 (m, 1H), 7.87 (m, 1H), 7.61 (dd, *J* = 2.1, 0.8 Hz, 1H), 7.55 (t, *J* = 7.9 Hz, 1H), 7.25 (dd, *J* = 2.2, 0.9 Hz, 1H), 5.50 – 5.38 (m, 1H), 4.89 – 4.63 (m, 1H), 4.14 – 3.77 (m, 1H), 3.75 – 3.30 (m, 3H), 3.29 – 3.21 (m, 2H), 2.53 (s, 3H), 2.46 (t, *J* = 7.0 Hz, 2H), 2.25 (m, 1H), 1.85 (m, 3H). ^{13}C NMR (100 MHz, CD_3CN) d 172.0 (d), 171.1 (d), 147.8, 142.2, 137.8, 133.7, 133.4, 132.3, 131.6, 129.3, 128.1, 127.9, 123.7, 119.6, 87.6 (dd), 78.9 (dd), 55.6, 45.0 (dd), 40.4 (d), 31.2 (d), 28.2 (dd), 19.4, 18.0. HRMS (TOF, ESI) calc'd for $C_{23}H_{23}BrClFN_2O_4S_2$ $[M+H]^+$ = 589.0028, found = 589.0026.

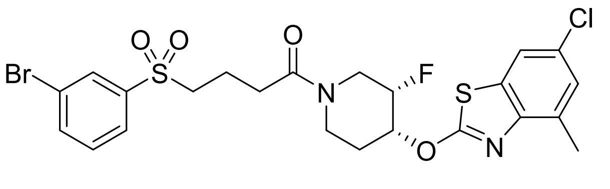

4-((3-Bromophenyl)sulfonyl)-1-((3*S*,4*R*)-4-((6-chloro-4-methylbenzo[*d*]thiazol-2-yl)oxy)-3-fluoropiperidin-1-yl)butan-1-one (VU6047608). Prepared in a similar manner as **VU6047606** using 4-(3-bromophenyl)sulfonylbutanoic acid (**17**, 50 mg, 0.16 mmol), 6-chloro-4-methyl-2-[(3*S*,4*R*)-3-fluoropiperidin-4-yl]oxy-1,3-benzothiazole (**12c**, 49 mg, 0.16 mmol), HATU (68.1 mg, 0.18 mmol), DIEA (42.5 µL, 0.24 mmol) and DCM (0.5 mL) to afford 42.4 mg of title compound (44% yield). ^{1}H NMR (400 MHz, CD_3CN) δ 8.04 (q, *J* = 1.5 Hz, 1H), 7.89 (m, 1H), 7.87 – 7.84 (m, 1H), 7.60 (d, *J* = 2.1 Hz, 1H), 7.55 (t, *J* = 7.9 Hz, 1H), 7.24 (dd, *J* = 2.2, 0.9 Hz, 1H), 5.49 – 5.33 (m, 1H), 5.23 – 5.00 (m, 1H), 4.60 – 3.97 (m, 1H), 4.40 – 3.72 (m, 1H), 3.51 – 2.86 (m, 4H), 2.53 (s, 3H), 2.51 – 2.40 (m, 2H), 2.12 – 1.98 (m, 2H), 1.92 – 1.83 (m, 2H). ^{13}C NMR (100 MHz, CD_3CN) δ 171.9 (d), 171.5 (d), 147.9, 142.2, 137.8, 133.6, 133.4, 132.3 (d), 131.6, 129.3, 128.0, 127.9, 123.7, 119.6, 86.9 (d), 79.1 (dd), 55.6, 45.9 (dd), 41.4 (d), 31.3 (d), 26.7 (dd), 19.5, 18.0. HRMS (TOF, ESI) calc'd for $C_{23}H_{23}BrClFN_2O_4S_2$ $[M+H]^+$ = 589.0028, found = 589.0032.

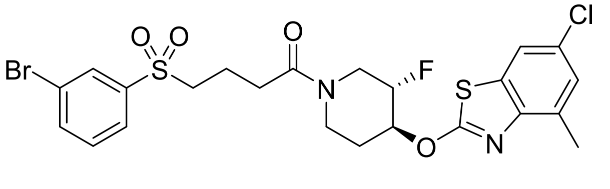

4-((3-Bromophenyl)sulfonyl)-1-((3*S*,4*S*)-4-((6-chloro-4-methylbenzo[*d*]thiazol-2-yl)oxy)-3-fluoropiperidin-1-yl)butan-1-one (VU6047609). Prepared in a similar manner as **VU6047606** using 4-(3-bromophenyl)sulfonylbutanoic acid (**17**, 50 mg, 0.16 mmol), 6-chloro-4-methyl-2-[(3*S*,4*S*)-3-fluoropiperidin-4-yl]oxy-1,3-benzothiazole (**12d**, 49 mg, 0.16 mmol), HATU (68.1 mg, 0.18 mmol), DIEA (42.5 µL, 0.24 mmol) and DCM (0.5 mL) to afford 50.2 mg of title compound (52% yield). ^{1}H NMR (400 MHz, CD_3CN) δ 8.04 (t, *J* = 1.9 Hz, 1H), 7.90 – 7.88 (m, 1H), 7.87 – 7.86 (m, 1H), 7.63 – 7.57 (m, 1H), 7.55 (t, *J* = 7.9 Hz, 1H), 7.27 – 7.22 (m, 1H), 5.50 – 5.38 (m, 1H), 4.89 – 4.62 (m, 1H), 4.17 – 3.75 (m,

1H), 3.75 – 3.31 (m, 3H), 3.29 – 3.21 (m, 2H), 2.53 (s, 3H), 2.46 (t, J = 6.9 Hz, 2H), 2.25 (m, 1H), 1.93 – 1.74 (m, 3H). ^{13}C NMR (101 MHz, CD_3CN) δ 172.0 (d), 171.1 (d), 147.9, 142.2, 137.8, 133.7, 133.4, 132.3, 131.6, 129.3, 128.0, 127.9, 123.7, 119.6, 87.6 (dd), 78.9 (dd), 55.6, 45.0 (dd), 40.4 (d), 31.2 (d), 28.0 (dd), 19.4, 18.0. HRMS (TOF, ESI) calc'd for $C_{23}H_{23}BrClFN_2O_4S_2$ $[M+H]^+$ = 589.0028, found = 589.0031.

Conditions Used for LC-MC/MS Analysis

Injection volume	10 µL	
Mobile phase A	0.5% Formic Acid in Water	
Mobile phase B	0.5% Formic Acid in Acetonitrile	
Flowrate	0.5 mL/min	
Gradient	Time	% Mobile Phase B
	0.0	5
	0.2	5
	0.8	95
	1.5	95
	1.7	5
	2.7	Stop
Column	Fortis C18 (50 x 3.0 mm, 3 µm)	
Data collection and analysis software/version	Analyst v. 1.7.1	
Ionization mode	Positive Electrospray	
Curtain gas (psi)	40	
GS1 (psi)	40	
GS2 (psi)	40	
Capillary voltage (V)	5500	
Source TurboIonSpray® temp. (°C)	500	

Ancillary Pharmacology

Table S1. Lead Profiling Screen – Eurofins Panlabs for **VU6032735**.

Assay Name	Species	% inhibition at 10 µM
Adenosine A_1	hum	12
Adenosine A_{2A}	hum	-6
Adenosine A_3	hum	9
Adrenergic α_{1A}	hum	-1
Adrenergic α_{1B}	hum	-5
Adrenergic α_{1D}	hum	2
Adrenergic α_{2A}	hum	-3
Adrenergic β_1	hum	8
Adrenergic β_2	hum	5
Androgen (Testosterone)	hum	1
Bradykinin B_1	hum	4
Bradykinin B_2	hum	-1
Calcium Channel L-Type, Benzothiazepine	rat	67
Calcium Channel L-Type, Dihydropyridine	rat	76
Calcium Channel N-Type	rat	2
Cannabinoid CB_1	hum	32
Dopamine D_1	hum	12
Dopamine D_{2S}	hum	-2
Dopamine D_3	hum	3
Dopamine $D_{4.4}$	hum	-5
Endothelin ET_A	hum	9
Endothelin ET_B	hum	1
Epidermal Growth Factor (EGF)	hum	5
Estrogen Erα	hum	11
$GABA_A$, Flunitrazepam, Central	rat	5
$GABA_A$, Muscimol, Central	rat	0
$GABA_{B1A}$	hum	31
Glucocorticoid	hum	10
Glutamate, Kainate	rat	-4
Glutamate, NMDA, Agonism	rat	-8
Glutamate, NMDA, Glycine	rat	-3
Glutamate, NMDA, Phencyclidine	rat	8
Histamine H_1	hum	14
Histamine H_2	hum	6
Histamine H_3	hum	-16

Imidazoline I_2, Central	rat	-2
Interleukin IL-1 R1	hum	-7
Leukotriene, Cysteinyl $CysLT_1$	hum	-3
Melatonin MT_1	hum	8
Muscarinic M_1	hum	-8
Muscarinic M_2	hum	-10
Muscarinic M_3	hum	7
Neuropeptide Y Y_1	hum	-1
Neuropeptide Y Y_2	hum	3
Nicotinic Acetylcholine α1, Bungarotoxin	hum	2
Nicotinic Acetylcholine α3β4	hum	-6
Opiate δ_1 (OP1, DOP)	hum	7
Opiate κ (OP2, KOP)	hum	-16
Opiate μ (OP3, MOP)	hum	11
Phorbol Ester	mouse	12
Platelet Activating Factor (PAF)	hum	-2
Potassium Channel [K_{ATP}]	ham	-7
Potassium Channel hERG, [^{3}H]Dofetilide	hum	9
Prostanoid EP_4	hum	-2
Purinergic P2X	rat	1
Purinergic P2Y, Non-Selective	rat	24
Rolipram	rat	7
Serotonin (5-Hydroxytryptamine) 5-HT_{1A}	hum	-1
Serotonin (5-Hydroxytryptamine) 5-HT_{2B}	hum	-6
Serotonin (5-Hydroxytryptamine) 5-HT_3	hum	-11
Sigma σ_1	hum	-3
Sodium Channel, Site 2	rat	81
Tachykinin NK_1	hum	7
Thyroid Hormone	rat	4
Transporter, Dopamine (DAT)	hum	26
Transporter, GABA	rat	-4
Transporter, Norepinephrine (NET)	hum	30

Table S2. Lead Profiling Screen – Eurofins Panlabs for **VU6047606**.

Assay Name	Species	% inhibition at 10 µM
Adenosine A_1	hum	5
Adenosine A_{2A}	hum	0
Adenosine A_3	hum	43
Adrenergic α_{1A}	hum	4
Adrenergic α_{1B}	hum	12
Adrenergic α_{1D}	hum	13
Adrenergic α_{2A}	hum	-4
Adrenergic β_1	hum	11
Adrenergic β_2	hum	5
Androgen (Testosterone)	hum	8
Bradykinin B_1	hum	-5
Bradykinin B_2	hum	4
Calcium Channel L-Type, Benzothiazepine	rat	49
Calcium Channel L-Type, Dihydropyridine	rat	93
Calcium Channel N-Type	rat	-1
Cannabinoid CB_1	hum	27
Dopamine D_1	hum	25
Dopamine D_{2S}	hum	-7
Dopamine D_3	hum	19
Dopamine $D_{4.4}$	hum	-8
Endothelin ET_A	hum	5
Endothelin ET_B	hum	9
Epidermal Growth Factor (EGF)	hum	2
Estrogen Erα	hum	6
$GABA_A$, Flunitrazepam, Central	rat	11
$GABA_A$, Muscimol, Central	rat	1
$GABA_{B1A}$	hum	12
Glucocorticoid	hum	18
Glutamate, Kainate	rat	-6
Glutamate, NMDA, Agonism	rat	-18
Glutamate, NMDA, Glycine	rat	-1
Glutamate, NMDA, Phencyclidine	rat	6
Histamine H_1	hum	11
Histamine H_2	hum	-24
Histamine H_3	hum	11
Imidazoline I_2, Central	rat	9
Interleukin IL-1 R1	hum	1
Leukotriene, Cysteinyl $CysLT_1$	hum	2

Melatonin MT_1	hum	19
Muscarinic M_1	hum	6
Muscarinic M_2	hum	-6
Muscarinic M_3	hum	13
Neuropeptide Y Y_1	hum	-16
Neuropeptide Y Y_2	hum	10
Nicotinic Acetylcholine α1, Bungarotoxin	hum	5
Nicotinic Acetylcholine α3β4	hum	-7
Opiate δ_1 (OP1, DOP)	hum	12
Opiate κ (OP2, KOP)	hum	-13
Opiate μ (OP3, MOP)	hum	22
Phorbol Ester	mouse	22
Platelet Activating Factor (PAF)	hum	8
Potassium Channel [K_{ATP}]	ham	-5
Potassium Channel hERG	hum	72
Prostanoid EP_4	hum	23
Purinergic P2X	rat	1
Purinergic P2Y, Non-Selective	rat	7
Rolipram	rat	19
Serotonin (5-Hydroxytryptamine) 5-HT_{1A}	hum	-2
Serotonin (5-Hydroxytryptamine) 5-HT_{2B}	hum	18
Serotonin (5-Hydroxytryptamine) 5-HT_3	hum	-17
Sigma σ_1	hum	62
Sodium Channel, Site 2	rat	83
Tachykinin NK_1	hum	8
Thyroid Hormone	rat	12
Transporter, Dopamine (DAT)	hum	18
Transporter, GABA	rat	4
Transporter, Norepinephrine (NET)	hum	44

Multi-species hepatocyte MetID

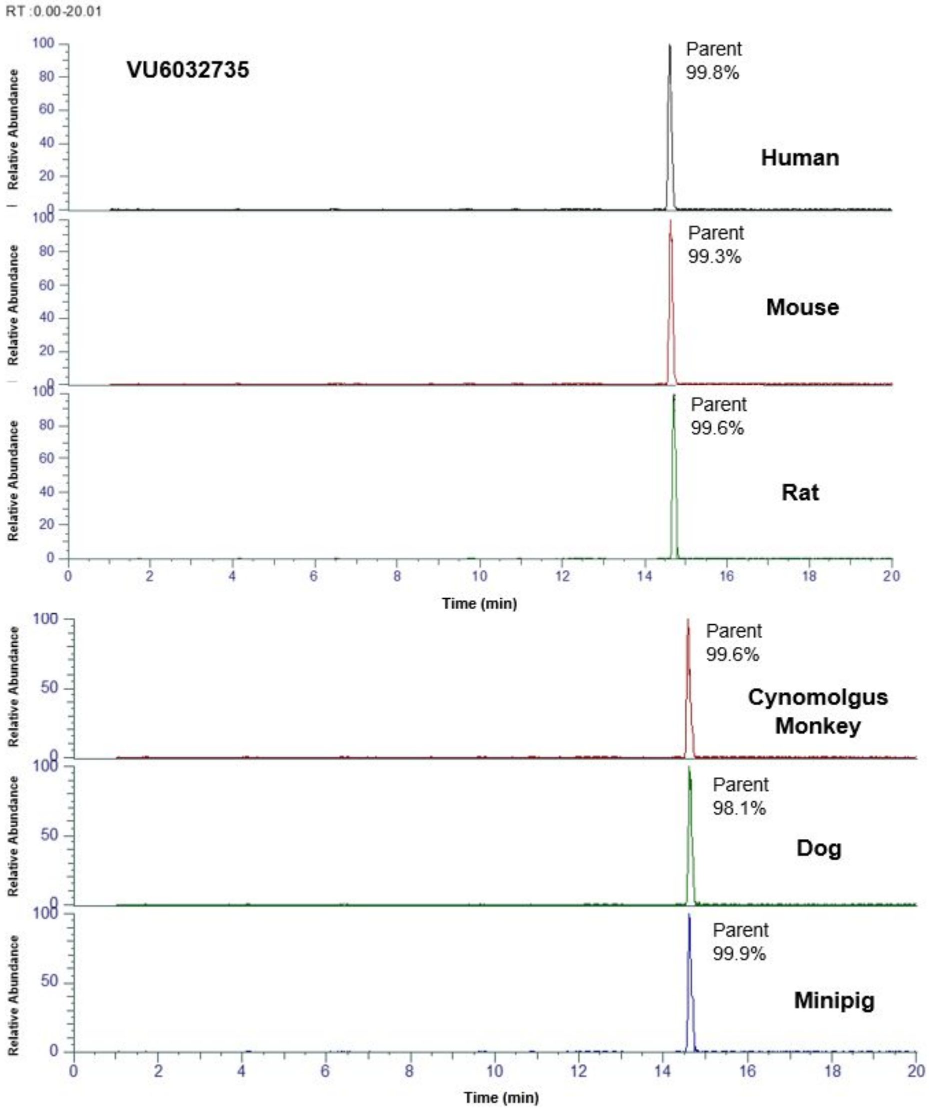

Figure S1. Multi-Species MetID studies show minimal metabolism of **VU6032735** after 4 hour incubation with hepatocytes.

Table S3. Multi-Species MetID study of **VU6047606** after 4-hour incubation with hepatocytes showing percentage of metabolites and parent compound remaining by species.

				% of Parent Related Material*					
**ID**	**Biotransformation**	**m/z**	**MW**	**Human**	**Cyno**	**Dog**	**Pig**	**Mouse**	**Rat**
**M781a**	**Hydroxylation, Glucoronidation**	**781.0294**	**780.0225**	**-**	**-**	**< 1**	**-**	**1.64**	**-**
**M781b**	**Hydroxylation, Glucoronidation**	**781.0301**	**780.0225**	**6.01**	**11.16**	**3.34**	**2.17**	**7.42**	**2.03**
**M604a**	**Hydroxylation**	**604.9961**	**603.9904**	**< 1**	**2.32**	**< 1**	**< 1**	**1.79**	**4.52**
**M604b**	**Hydroxylation**	**604.9999**	**603.9904**	**1.42**	**2.72**	**1.20**	**1.46**	**< 1**	**< 1**
**M604c**	**Hydroxylation**	**604.9995**	**603.9904**	**< 1**	**< 1**	**-**	**< 1**	**< 1**	**< 1**
**M604d**	**Hydroxylation**	**604.9980**	**603.9904**	**< 1**	**< 1**	**< 1**	**1.72**	**2.87**	**< 1**
**Parent**	**VU6047606**	**589.0037**	**587.9955**	**91.66**	**82.77**	**93.78**	**93.66**	**84.99**	**90.93**

Inhibition of human SLO3-γ2 currents in HEK293 cells.

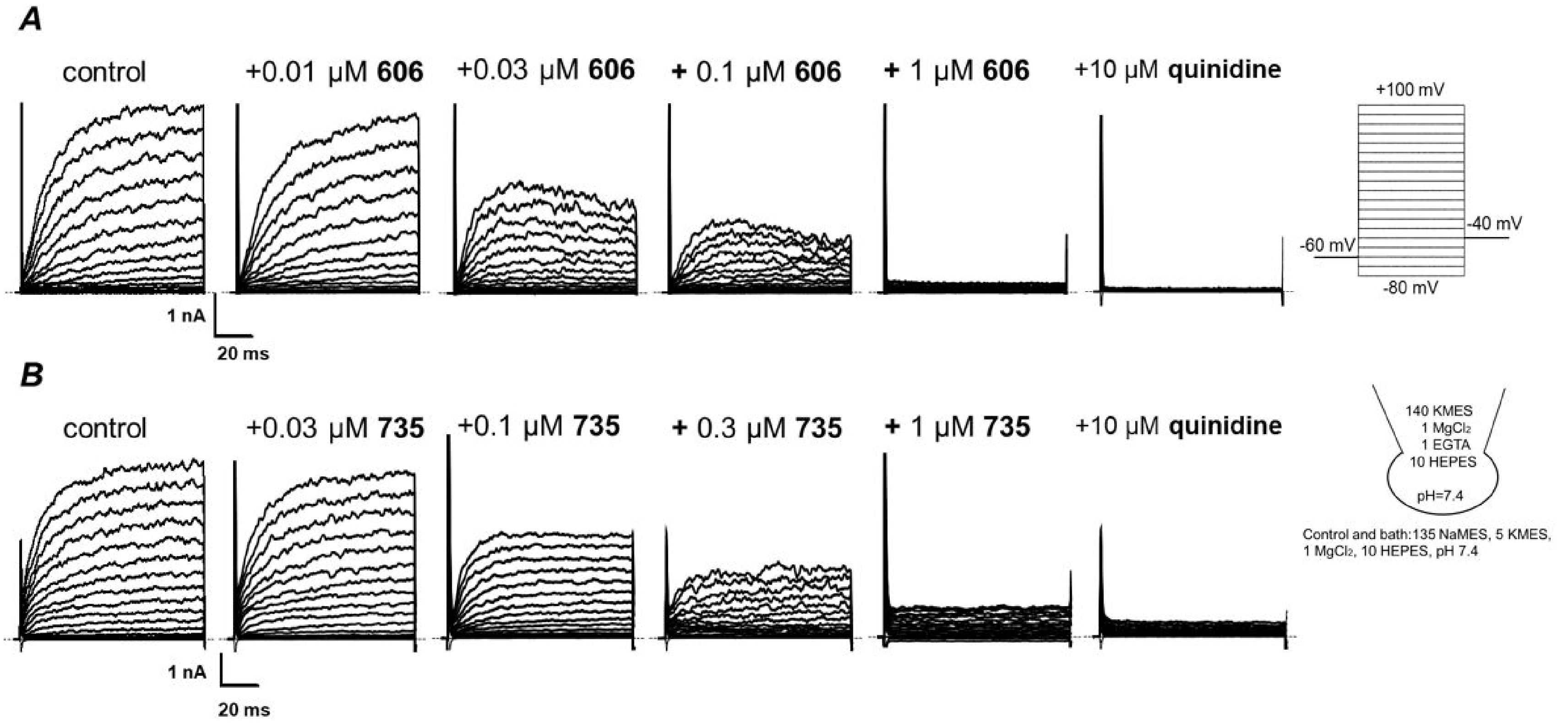

Figure S2. Inhibition of Human SLO3-γ2 Channels by VU6047606 and VU6032735 in HEK293 Cells. Representative whole-cell current recordings at indicated concentrations of **VU6047606** (***A***) or **VU6032735** (***B***). To confirm hSLO3-γ2 channel expression, 10 μM quinidine was applied at the end of the recording. The right panel illustrates the whole-cell patch-clamp configuration along with intra- and extracellular ionic compositions. hSLO3-γ2 Currents were elicited from a Vh= -60 mV with voltage steps from -80 to +100 mV in 10 mV steps.

<u>Inhibition of mouse SLO3-γ2 currents in HEK293 cells.</u>

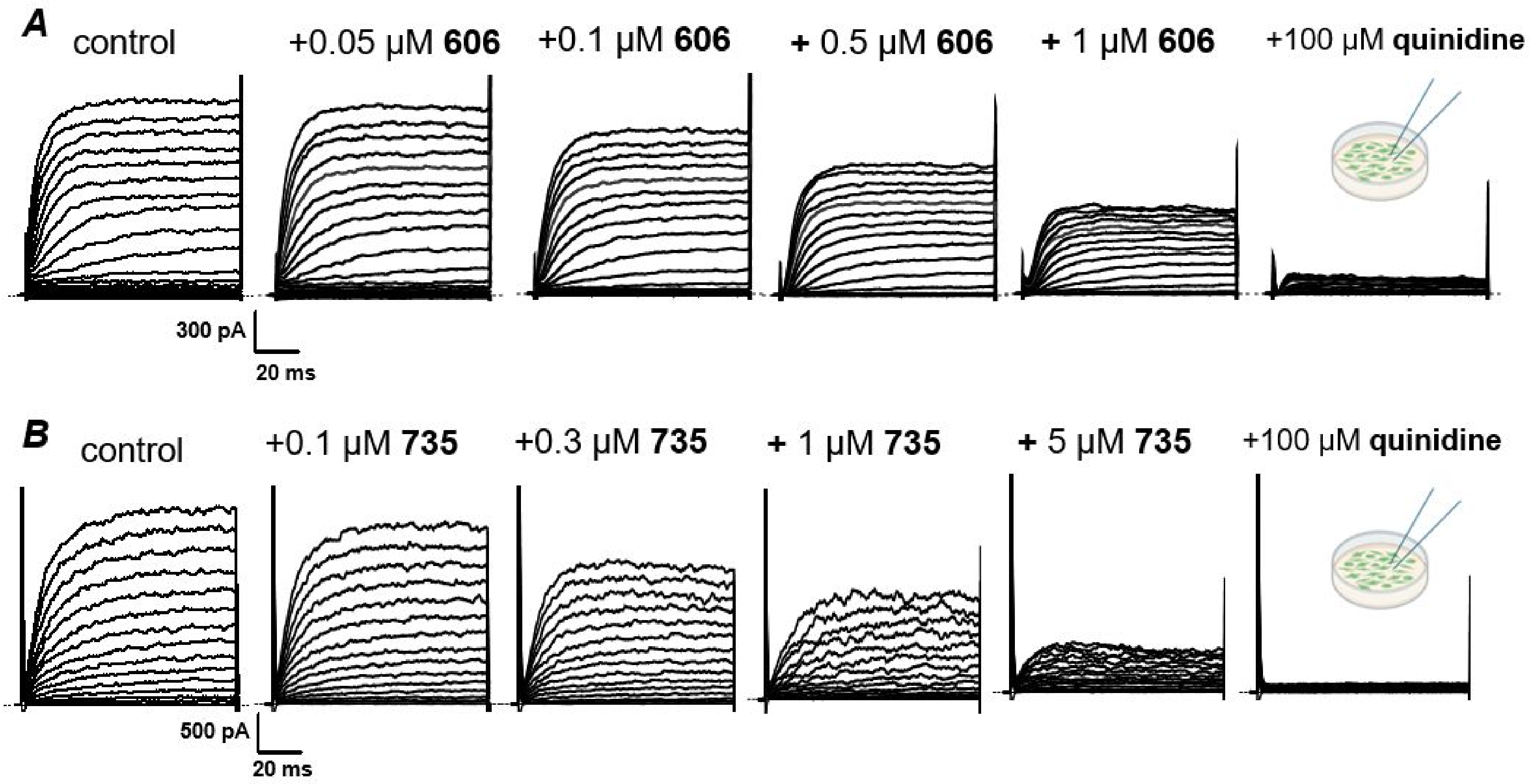

Figure S3. VU6047606 and VU6032735 inhibit mouse SLO3-γ2 channels in HEK293 cells. Representative traces of mSLO3-γ2 currents at multiple indicated concentrations of **VU6047606** (***A***) or **VU6032735** (***B***). 100 μM quinidine was applied by the end of recording to confirm the SLO3-γ2 expression.

Inhibition of KSper currents in mouse sperm.

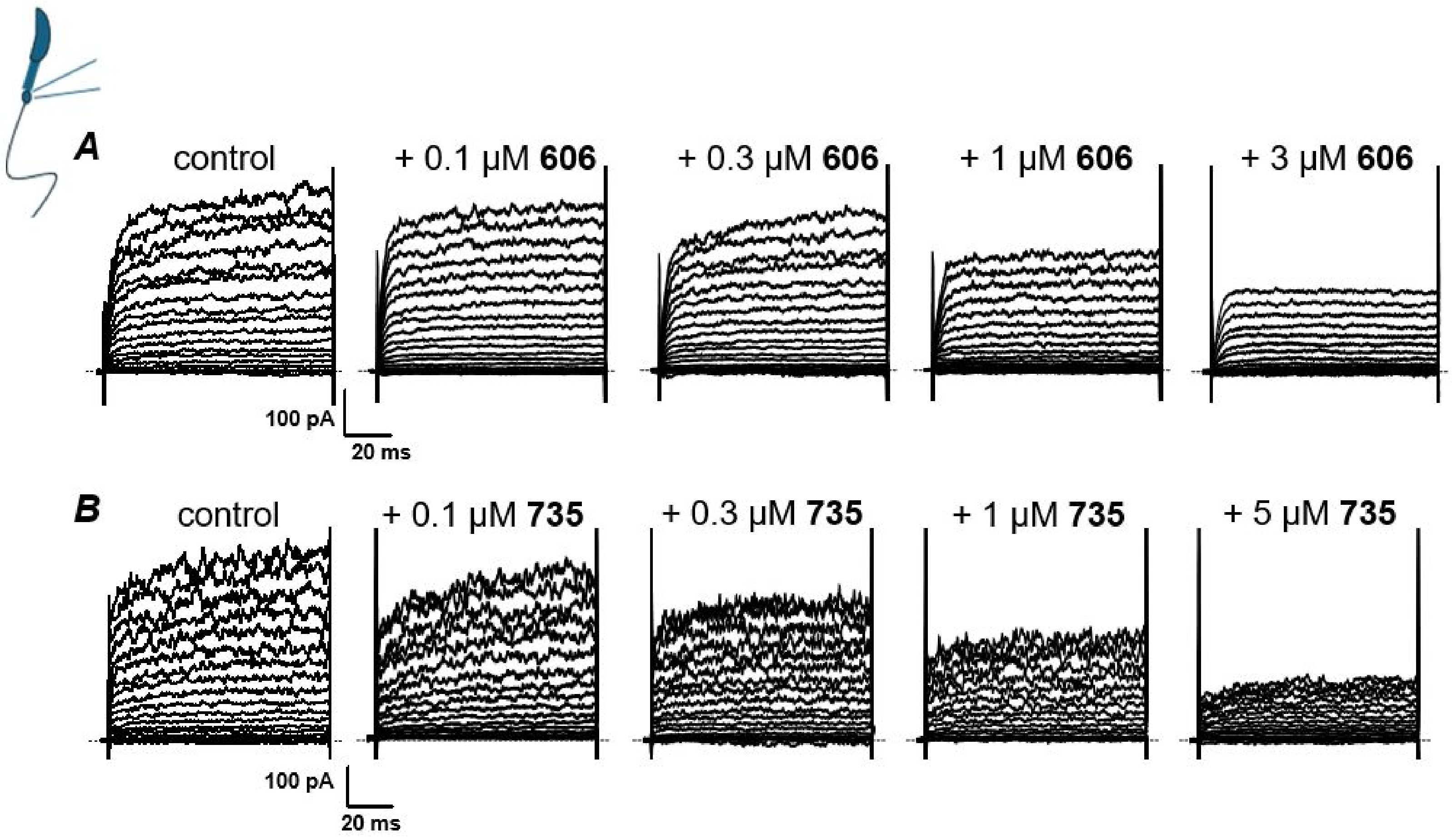

Figure S4. VU6047606 and VU6032735 inhibit KSper currents in mouse sperm. Representative traces of KSper currents at multiple indicated concentrations of **VU6047606** (*A*) or **VU6032735** (***B***).
